# The First Total Synthesis of (±)-Methyl Salvianolate A Using a Convergent Strategy

**DOI:** 10.3390/molecules24050999

**Published:** 2019-03-12

**Authors:** Bo Wang, Liping Wang, Ying Peng, Yiying Pang, Hesheng Xiao, Xiaoji Wang, Shuangping Huang

**Affiliations:** 1 School of Chemistry and Chemical Engineering, Jiangxi Science and Technology Normal University, Nanchang 330013, China; wbo0728@163.com; 2 School of Life Science, Jiangxi Science and Technology Normal University, Nanchang 330013, China; wlping92@163.com (L.W.); dapengpeng@163.com (Y.P.); 13667080401@163.com (Y.P.); xiaohes93@163.com (H.X.); 3 School of Pharmacy, Jiangxi Science and Technology Normal University, Nanchang 330013, China; 4 College of Biomedical Engineering, Taiyuan University of Technology, Taiyuan 030024, China

**Keywords:** methyl salvianolate A, Danshen, total synthesis, Horner–Wadsworth–Emmons reaction, silyl protecting group

## Abstract

Herein, a convergent, practicable and first total synthesis of the natural product, (±)-methyl salvianolate A, is reported. The key features of the approach are the use of a Horner–Wadsworth–Emmons reaction and the protection of multiple hydroxyls using silyl protecting groups. The employment of the readily removable silyl protecting groups allows the synthesis of (±)-methyl salvianolate A and its derivatives on a reasonably large scale.

## 1. Introduction

**Figure 1:**
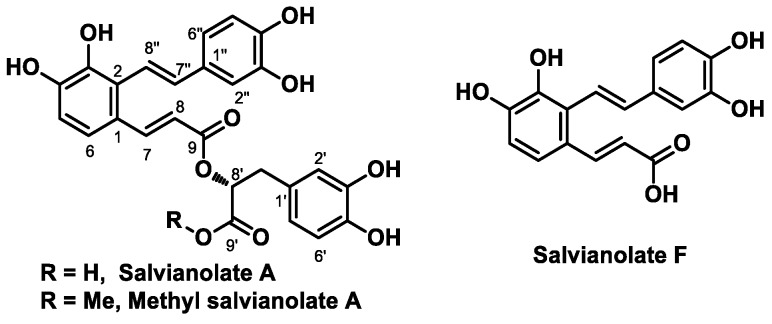
The structures of salvianolate F and A, and (±)-methyl salvianolate A.

Traditional Chinese medicines, which are rich in bioactive natural products, and are widely used in China and Asian countries, play a significant role in the treatment of several diseases. The Chinese herb Danshen from the Chinese Pharmacopoeia has been widely used, for example, to treat cerebro-cardiovascular, gynecologic, and digestive diseases [1]. The significant pharmacological activity may be attributed to the novel biological active components present in Danshen. Phenolic acids are one of the most important types of water-soluble bioactive components. In 1984, Li and co-workers first isolated the polyphenol acid, (+)-salvianolate A (Figure 1), from the root of Danshen [2]. Salvianolate A showed considerable biological properties, including antioxidant [3,4,5,6], cardio- protective [7,8], antiplatelet, and antithrombotic activities [9], as well as reversing the paclitaxel resistance and inhibiting tumor migration and invasion of human breast cancer [10]. These multiple pharmacological effects attracted numerous pharmacological groups to devote their research efforts toward investigating salvianolate A. Twenty three years later, its methyl derivative, (±)-methyl salvianolate A, was first isolated by the Chen’s group [11] from the aqueous extracts of the roots of another species of the *Salvia* genus, *Salvia yunnanensis*, and was found to possess pharmacological effects similar to those of salvianolate A, including anti-HIV-1 activity. Due to its similar structure to salvianolate A, (±)-methyl salvianolate A may also possess the other pharmacological effects than those displayed by salvianolate A. However, no attention has been directed toward the synthesis of (±)-methyl salvianolate A. Despite the numerous biological studies conducted on salvianolate A, only four total syntheses of salvianolate A [12,13,14,15] and one for salvianolate F [16], which possesses a similar structure to (±)-methyl salvianolate A, have been reported to date. Considering the excellent activity and potential pharmacological applications of (±)-methyl salvianolate A, but its low concentration in *Salvia* spp. tissues, we became interested in developing an efficient approach towards the synthesis of (±)-methyl salvianolate A. Herein, we describe the first total synthesis of (±)-methyl salvianolate A, using a convergent approach [17].

## 2. Results

**Scheme 1:**
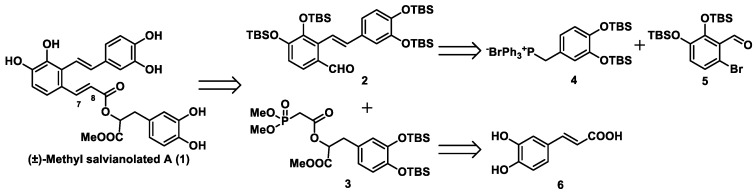
Retrosynthetic analysis of (±)-methyl salvianolate A.

To the best of our knowledge, most of the published formal syntheses of polyphenol compounds that are structurally similar to (±)-methyl salvianolate A involve the use of methyl ether protecting groups, which effectively avoids the influence of the phenol groups during the synthesis. However, the use of the methyl ether protecting group results in significant side reactions and low yields during its deprotection, in which a strong Lewis acid, such as BBr_3_, is usually used. Although an allylic protective group which is more easily removed was employed by Zhang’s group, only a moderate yield (41%) was achieved under the deprotection conditions (Pd(PPh_3_)_4_, morpholine) [15]. As an alternative to using an alkyl group, we looked to install the readily removable tert-butyldimethylsilyl (TBS) group as the protecting group in our total synthesis. Our retrosynthetic analysis of methyl salvianolate A is shown in Scheme 1. (±)-Methyl salvianolate A was disconnected at the C7-C8 bond via a Horner–Wadsworth–Emmons (HWE) reaction to give two Fragments **2** and **3**. We envisaged that fragment **2** could be obtained from fragments **4** and **5** via a Wittig reaction using Cotelle’s protocol [16], and that fragment **3** could be prepared from caffeic acid (fragment **6**).

**Scheme 2:**
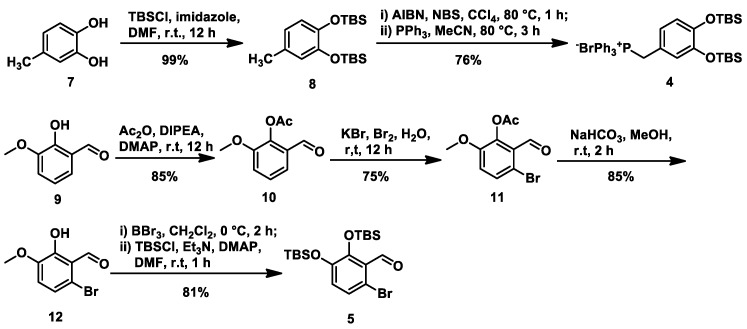
Synthesis of intermediates **4** and **5**.

As depicted in Scheme 2, our synthesis of (±)-methyl salvianolate A commenced with the preparation of intermediate **4** from commercially available 4-methylcatechol (**7**). 4-Methylcatechol was subjected to TBS protection under conventional conditions (TBSCl/imidazole in DMF) to give **8** in an almost quantitative yield. Compound **8** was then subjected to bromination at the benzylic position via a radical process using *N*-bromosuccinimide (NBS) in the presence of 2,2′-azobis- (isobutyronictrile) (AIBN) in CCl_4_ at 80 °C. Without further purification, the resultant bromide was reacted with triphenyl phosphine in anhydrous MeCN at 80 °C, followed by washing with toluene to give phosphonium salt **4** in an overall 76% yield from **8**. Meanwhile, intermediate **5** was prepared from commercially available *o*-vanillin (**9**). To avoid the influence of the free phenol hydroxyl group, aldehyde **9** first reacted with acetic anhydride to afford **10**, which was then converted to monobromide **11** in the presence of potassium bromide and bromine in water (64% over two steps). The acetyl group in **11** was removed under mild reaction conditions (sodium hydrogen carbonate in MeOH) followed by removal of the methyl group using BBr_3_ at 0 °C and a second protection step using TBSCl in the presence of triethylamine and 4-dimethylaminopyridine (DMAP) to achieve intermediate **5** in 69% yield over three steps.

**Scheme 3:**
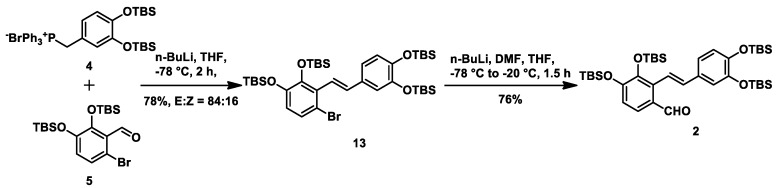
Synthesis of fragment **2**.

With intermediates **4** and **5** in hand, we then conducted the Wittig reaction. Treating salt **4** with n-BuLi gave the corresponding ylide that was then reacted with aldehyde **5** at −78 °C to afford alkene **13** in 78% yield with a *E/Z* ratio of 84:16. On exposure to *n*-BuLi at −78 °C, the resulting anion prepared in situ from the lithium-halogen exchange of alkene **13** was reacted with *N*,*N*-dimethylformamide at −20 °C to give aryl aldehyde **2** in 76% yield (Scheme 3).

**Scheme 4:**
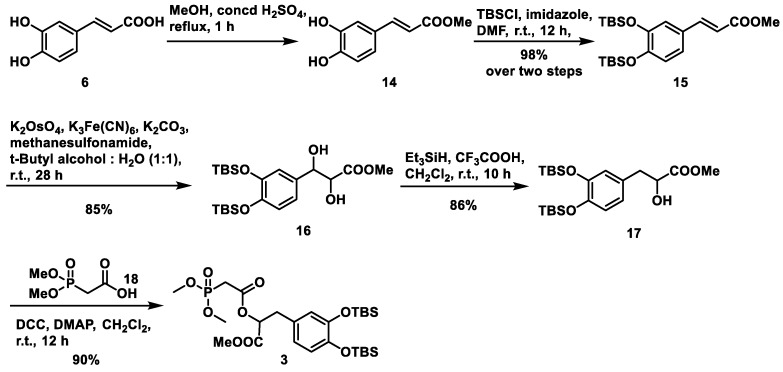
Synthesis of fragment **3**.

For fragment **3**, we envisaged an esterification reaction between the commercially available acid **18** and alcohol **17**. The latter was synthesized following an established procedure [18] starting from caffeic acid **6**, as shown in Scheme 4, which included esterification, TBS protection, dihydroxylation and selective dehydroxylation steps. We obtained intermediate **17** in an overall 72% yield from **6**. Subsequently, when treated with *N*,*N*′-dicyclohexylcarbodiimide (DCC) and catalytic DMAP in CH_2_Cl_2_ at room temperature, **17** and **18** were assembled into fragment 3 in 90% yield.

**Scheme 5:**
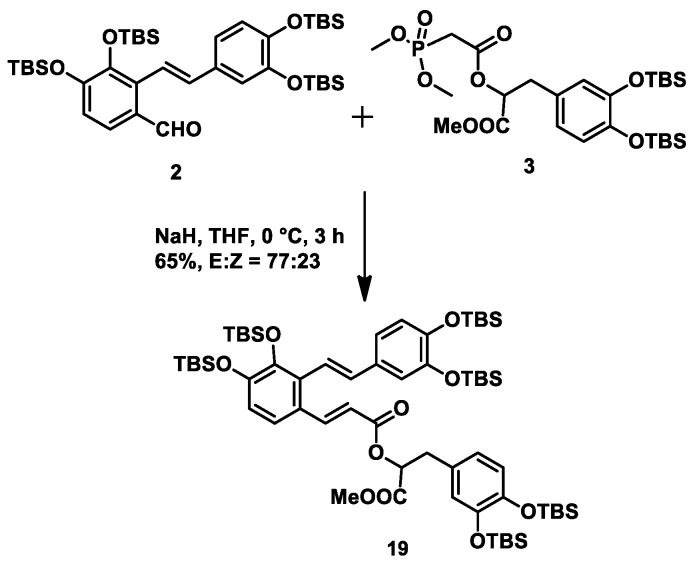
Assembly of intermediate **19** via HWE reaction.

At this stage, we needed to connect fragments **2** and **3** to construct the α,β-unsaturated ester moiety via a HWE reaction. After conducting some experiments in different conditions (*n*-BuLi, −78 °C; NaH, −78 °C; NaH, 0 °C), we successfully obtained intermediate **19** in 65% yield with an *E/Z* ratio of ~4:1 when performing the HWE reaction at 0 °C for 3 h using NaH as base (Scheme 5).

Finally, with **19** in hand, we turned to conduct the global deprotection step according to our retrosynthetic analysis. However, the deprotection proved surprisingly difficult. It was disappointing to find that the desired product was not obtained when treating **19** with the conventional reagent, tetrabutylammonium fluoride (TBAF). A great deal of TBAF and silicide accompanied **1** during the purification step, which hindered the isolation of the target product (Table 1, Entry 1).

A similar phenomenon was observed when conducting the experiment under basic [19,20,21] or acidic conditions, it gave either no reaction (Entry 2–4,6) or trace amounts of the isolated purified product mixed with a large amount of silicide (Entry 5,7,8). Fortunately, we finally obtained (±)-methyl salvianolate A (1) in excellent yield (92%) without any silicide after purification using triethylamine trihydrofluoride in anhydrous pyridine (Entry 9) [18,22].

Considering the low efficiency of the demethylation step in the endgame of the formally published total syntheses of the salvianolates, the deprotection of 19 employing the readily removable silyl ether protection group demonstrated that (±)-methyl salvianolate A and its derivatives can be prepared on a large scale using our strategy. The physical properties and ^1^H- and ^13^C-NMR spectra of compound 1 obtained using our synthetic route agreed with those reported in the literature [11].

## 3. Experimental Section

### 3.1. General Methods

All air and water sensitive reactions were carried out under a nitrogen atmosphere with dry solvents under anhydrous conditions, unless otherwise noted. Reactions were monitored by thin-layer chromatography (TLC) carried out on 0.25 mm silica gel plates (60F-254, Qingdao Ocean Chemical Factory, Qingdao, China) that were analyzed by fluorescence upon 254 nm irradiation or by staining with anisaldehyde (450 mL of 95% EtOH, 25 mL of con. H_2_SO_4_ (15 mL) of acetic acid, and 25 mL of anisaldehyde, phosphomolybdic acid (100 mL of 95% EtOH, 10 g phosphomolybdic acid) and dinitrophenylhydrazine (80 mL H_2_O, 200 mL of 95% EtOH, 12 g dinitrophenylhydrazine and 60 mL concentrated sulfuric acid). Silica gel (60, particle size 0.040–0.063 mm) was used for flash column chromatography. All the chemicals were purchased commercially and used without further purification. Anhydrous THF was distilled from sodium-benzophenone. DMF, CH_3_CN and CH_2_Cl_2_ were distilled from calcium hydride. Yields refer to chromatographically, unless otherwise stated. NMR spectra were recorded on an Avance 400 MHz instrument (^1^H: 400 MHz, ^13^C: 100 MHz, Bruker Corporation, Karlsruhe, Germany). The following abbreviations were used to explain themultiplicities: s = singlet, d = doublet, m = multiplet. High-resolution mass spectra were obtained from a MALDI-TOF Mass Spectrometer (Applied Biosystems, Carlsbad, CA, USA). IR spectra were obtained using FT-IR Spectrometer (Bruker Vertex 70, Bruker Corporation, Karlsruhe, Germany). NMR and HRMS of compounds **1–5, 8, 10–17, 19** can be found in Supplementary Materials.

### 3.2. 3,4-Bis(tert-butyldimethylsilyloxy)toluene *(**8**)*

Imidazole (13.7 g, 201.39 mmol) and *tert*-butyldimethylchlorosilane (18.2 g, 120.83 mmol) were added to a solution of **7** (5.0 g, 40.28 mmol), which was dissolved in dry DMF (200 mL), at 0 °C under a nitrogen atmosphere. The resulting mixture was warmed to room temperature and stirred overnight. The reaction was quenched with brine, extracted with ethyl acetate, and the combined organic layers were washed with brine, dried over anhydrous sodium sulfate, filtered and concentrated in vacuo. The resulting residue was purified by silica gel chromatography to afford compound 8 (14.20 g, 99%) as a colorless liquid. ^1^H-NMR (CDCl_3_): δ 6.72 (d, *J* = 8.0 Hz, 1H), 6.65 (s, 1H), 6.61 (d, *J* = 8.0 Hz, 1H), 2.24 (s, 3H), 1.01 (s, 9H), 1.00 (s, 9H), 0.21 (s, 6H), 0.20 (s, 6H).; HRMS (TOF-MS): *m*/*z* calcd for C_19_H_37_O_2_Si_2_ [M + H]^+^ 353.2327. Found 353.2327.

### 3.3. (3,4-Bis((tert-butyldimethylsilyl)oxy)benzyl)triphenylphosphonium Bromide *(**4**)*

Under a nitrogen atmosphere, *N*-bromosuccinimide (7.28 g, 40.83 mmol) and AIBN (1.1 g, 6.81 mmol) were added to a solution of compound **8** (12.0 g, 34.03 mmol) in CCl_4_ (70 mL) and the resulting mixture heated at reflux for 1 h. The reaction mixture was filtered and the residue was washed with petroleum ether and concentrated in vacuo to afford the crude product, which was immediately used in the next step without any purification. Triphenylphosphine (13.38 g, 51.04 mmol) was then added to the crude product solution in dry MeCN (70 mL) and the resulting mixture was heated under reflux for 3 h. The reaction mixture was cooled to room temperature and concentrated in vacuo. Toluene was added to the crude product and the resulting suspension filtered to give phosphonium salt **4** (17.94 g, 76% over two steps) as a pale yellow solid. ^1^H-NMR (CDCl_3_): δ 7.79–7.62 (m, 15H), 6.61 (s, 2H), 6.47 (s, 1H), 5.16 (d, *J* = 13.6 Hz, 2H), 0.93 (s, 9H), 0.84 (s, 9H), 0.15 (s, 6H), −0.05 (s, 6H); HRMS (TOF-MS): *m*/*z* calcd for C_37_H_50_O_2_Si_2_P [M-Br^-^]^+^ 613.3081. Found 613.3082.

### 3.4. 2-Formyl-6-methoxyphenyl Acetate *(**10**)*

A catalytic quantity of 4-dimethylaminopyridine (0.96 g, 7.89 mmol) and acetic anhydride (8.07 mL, 85.44 mmol) were added to an *o*-vanillin (**9**, 10.01 g, 65.72 mmol) solution in *N*,*N*-diisopropylethylamine (33 mL) at 0 °C under a nitrogen atmosphere. The solution was allowed to warm to room temperature and stirred overnight. After compound 9 was completely consumed, hydrochloric acid (2 M, 100 mL) was added. The resulting mixture was stirred for another 10 min and then extracted with dichloromethane (100 mL × 3). The combined organic layers were concentrated in vacuo to afford a crude yellow solid, which was recrystallized from ethanol to give compound **10** (10.85 g, 85%) as a yellow solid. ^1^H-NMR (CDCl_3_): δ 10.12 (s, 1H), 7.43 (d, *J* = 7.6 Hz, 1H), 7.31 (t, *J* = 8.4 Hz, 1H), 7.20 (d, *J* = 8.4 Hz, 1H), 3.85 (s, 3H), 2.39 (s, 3H).; ^13^C-NMR (100 MHz, CDCl_3_): δ 188.7, 168.7, 151.8, 141.5, 129.2, 126.8, 121.2, 117.9, 56.3, 20.4.

### 3.5. 3-Bromo-2-formyl-6-methoxyphenyl Acetate *(**11**)*

Bromine (3.73 mL, 72.64 mmol) was added to a potassium bromide (21.47 g, 180.48 mmol) solution in H_2_O (133 mL). Aldehyde **10** (10.85 g, 55.88 mmol) was added to the resulting dark red solution and stirred overnight. The reaction was monitored by TLC until **10** was completely consumed. The reaction mixture was extracted with ethyl acetate, the layers separated and the organic layer concentrated in vacuo. The resulting residue was recrystallized from ethyl acetate–hexane to give compound **11** (11.44 g, 75%) as yellow crystals. ^1^H-NMR (CDCl_3_): δ 10.35 (s, 1H), 7.59 (d, *J* = 8.8 Hz, 1H), 7.14 (d, *J* = 8.8 Hz, 1H), 3.94 (s, 3H), 2.47 (s, 3H); ^13^C-NMR (CDCl_3_): δ 190.3, 168.7, 151.9, 140.4, 131.5, 126.6, 117.8, 116.3, 56.4, 20.5.

### 3.6. 6-Bromo-2-hydroxy-3-methoxybenzaldehyde *(**12**)*

Sodium bicarbonate (4.93 g, 58.64 mmol) was added to a solution of aldehyde **11** (11.44 g, 41.89 mmol) solution in a mixture of methanol and water (*v/v* = 2:1, 84 mL). The resulting turbid yellow solution was stirred for 2 h at room temperature. Hydrochloric acid (1 M) was then added to adjust the pH to 6–7 and the mixture extracted with ethyl acetate (100 mL × 3). The combined organic layers were concentrated in vacuo and the crude yellow solid recrystallized from ethyl acetate/hexane to give compound **12** (8.23 g, 85%) as yellow acicular crystals. ^1^H-NMR (CDCl_3_): δ 12.26 (s, 1H), 10.27 (s, 1H), 7.07 (d, *J* = 8.4 Hz, 1H), 6.90 (d, *J* = 8.4 Hz, 1H), 3.88 (s, 3H); ^13^C-NMR (CDCl_3_): δ 198.3, 154.4, 148.3, 123.3, 118.2, 117.1, 116.3, 56.3.

### 3.7. 6-Bromo-2,3-bis((tert-butyldimethylsilyl)oxy)benzaldehyde *(**5**)*

Boron tribromide (13.47 mL, 142.48 mmol) in CH_2_Cl_2_ (60 mL) was added dropwise to a solution of compound **12** (8.23 g, 35.62 mmol) in dry CH_2_Cl_2_ (120 mL) at 0 °C under a nitrogen atmosphere. The resulting mixture was then stirred for 2 h. Ice–water (100 mL) was added slowly to quench the reaction, the layers separated and the organic layer concentrated in vacuo. The residue was washed with water, filtered and the resulting yellow filter cake dried at 40 °C to give the crude product as a yellow solid, which was used in the next step without any further purification. Et_3_N (16.50 mL, 118.37 mmol), DMAP (0.826 g, 6.76 mmol) and *tert*-butyldimethylchlorosilane (15.29 g, 101.46 mmol) were added to the above crude solid (7.34 g, 33.82 mmol) in dry DMF (307 mL) at 0 °C under a nitrogen atmosphere. The resulting solution was stirred for 1 h at 0 °C. The reaction was quenched with brine, extracted with ethyl acetate (200 mL × 3) and the combined organic layers concentrated in vacuo. The resulting residue was purified by silica gel chromatography to give intermediate 5 (12.85 g, 81% over two steps). ^1^H-NMR (CDCl_3_): δ 10.29 (s, 1H), 7.11 (d, *J* = 8.4 Hz, 1H), 6.87 (d, *J* = 8.8 Hz, 1H), 1.00 (s, 9H), 0.96 (s, 9H), 0.23 (s, 6H), 0.12 (s, 6H); ^13^C-NMR (CDCl_3_): δ 189.4, 149.3, 146.9, 127.0, 126.0, 124.6, 112.8, 25.1, 25.0, 17.7, 17.5, −4.7, −4.8; HRMS (TOF-MS): *m/z* calcd for C_19_H_34_BrO_3_Si_2_ [M + H]^+^ 445.1224. Found 445.1230.

### 3.8. (E)-6-Bromo-2,3,3’,4’-tetrakis(tert-butyldimethylsilyloxy)stilbene *(**13**)*

*n*-BuLi (10.3 mL, 2.5 M in hexane, 25.81 mmol) was slowly added to a suspension of phosphonium salt **4** (19.47 g, 28.06 mmol) in dry THF (100 mL) at –78 °C under a nitrogen atmosphere and the resulting mixture was stirred for 30 min at –78 °C. Aldehyde **5** (5.0 g, 11.22 mmol) dissolved in dry THF (40 mL) was added dropwise to the reaction mixture and the resulting solution stirred for another 1 h at –78 °C. The reaction was quenched with a saturated aqueous solution of ammonium chloride. The mixture was extracted with ethyl acetate (100 mL × 3) and the combined organic layers washed with brine, dried over anhydrous sodium sulfate, filtered and concentrated in vacuo. The resulting residue was purified by silica gel chromatography to give compound **13** (17.08 g, 78%, *E*:*Z* = 84:16) as a colorless oil. ^1^H-NMR (CDCl_3_): δ 7.11 (d, *J* = 8.4 Hz, 1H), 7.02 (d, *J* = 8.8 Hz, 1H), 7.00 (d, *J* = 17.2 Hz, 1H), 6.94 (s, 1H), 6.85 (d, *J* = 16.8 Hz, 1H), 6.84 (d, *J* = 8.4 Hz, 1H), 6.66 (d, *J* = 8.4 Hz, 1H), 1.04 (s, 18H), 1.02 (s, 9H), 0.95 (s, 9H), 0.28 (s, 6H), 0.26 (s, 12H), 0.09 (s, 6H).; ^13^C-NMR (CDCl_3_): δ 147.3, 146.9, 146.8, 145.6, 135.2, 131.8, 131.2, 125.4, 123.5, 121.0, 120.0, 119.1, 115.5, 26.2, 26.2, 26.0, 25.9, 18.8, 18.5, 18.5, 18.3, −3.5, −3.6, −4.0 −4.1; HRMS (TOF-MS): *m*/*z* calcd for C_36_H_68_BrO_4_Si_4_ [M + H]^+^ 779.3373. Found 779.3378.

### 3.9. (E)-2-(3,4-Bis((tert-butyldimethylsilyl)oxy)styryl)-3,4-bis((tert-butyldimethyl-silyl)oxy)benzaldehyde *(**2**)*

*n*-BuLi (6.7 mL, 2.5 M in hexane, 16.82 mmol) was added to a solution of compound **13** (6.56 g, 8.41 mmol) in dry THF (40 mL) at −78 °C under a nitrogen atmosphere. After the reaction mixture was stirred for 30 min at −78 °C, dry *N*,*N*-dimethylformamide (6.50 mL, 84.08 mmol) in dry THF (10 mL) was slowly added to the mixture and stirred for another 30 min at −78 °C until **13** was completely consumed. The reaction was quenched with a saturated aqueous solution of ammonium chloride and hydrochloric acid (1 M) added to adjust the pH to 1. The mixture was extracted with ethyl acetate (50 mL × 3) and the combined organic layers dried over anhydrous Na_2_SO_4_, filtered and concentrated in vacuo. The resulting residue was purified by silica gel chromatography to give compound **2** (4.66 g, 76%) as a yellow oil. ^1^H-NMR (CDCl_3_): δ 10.08 (s, 1H), 7.56 (d, *J* = 8.4 Hz, 1H), 7.18 (d, *J* = 16.4 Hz, 1H), 7.02 (s, 1H), 6.99 (d, *J* = 8.4 Hz, 1H), 6.91 (d, *J* = 8.4 Hz, 1H), 6.85 (d, *J* = 8.0 Hz, 1H), 6.46 (d, *J* = 16.4 Hz, 1H), 1.03 (s, 9H), 1.03 (s, 9H), 1.02 (s, 9H), 0.99 (s, 9H), 0.31 (s, 6H), 0.26 (s, 6H), 0.25 (s, 6H), 0.13 (s, 6H).; ^13^C-NMR (CDCl_3_): δ 192.6, 152.9, 148.4, 147.9, 145.5, 139.4, 137.3, 131.4, 130.2, 124.0, 122.0, 121.4, 120.5, 120.4, 120.2, 27.1, 27.0, 26.9, 19.8, 19.4, 19.4, 19.3, −2.4, −2.6, −3.1, −3.1; HRMS (TOF-MS): *m*/*z* calcd for C_39_H_69_O_5_Si_4_ [M + H]^+^ 729.4217. Found 729.4218.

### 3.10. (E)-methyl 3-(3,4-dihydroxyphenyl)acrylate *(**14**)*

Concentrated sulfuric acid (4 mL) was slowly added to a solution of caffeic acid (**6**, 2.0 g, 11.10 mmol) in methanol (140 mL). The resulting mixture was heated at reflux for 1 h. Aqueous NaOH (1 M) was added to quench the reaction and adjust the pH to 6–7. The aqueous phase was extracted with CH_2_Cl_2_ (100 mL × 3). The combined organic layers were washed with brine, dried over anhydrous sodium sulfate, filtered and concentrated in vacuo. The resulting crude product (2.15 g) was used in the next step without any further purification. ^1^H-NMR (CD_3_OD): δ 7.51 (d, *J* = 16.0 Hz, 1H), 7.03 (s, 1H), 6.90 (d, *J* = 8.0 Hz, 1H), 6.77 (d, *J* = 8.4 Hz, 1H), 6.22 (d, *J* = 16.0 Hz, 1H), 4.98 (br s, 2H), 3.71 (s, 3H).; ^13^C-NMR (CD_3_OD): δ 167.5, 147.1, 144.6, 144.4, 125.5, 120.7, 114.2, 113.0, 112.6, 49.8; HRMS (TOF-MS): *m*/*z* calcd for C_10_H_11_O_4_ [M + H]^+^ 195.0652. Found 195.0658.

### 3.11. (E)-methyl 3-(3,4-bis((tert-butyldimethylsilyl)oxy)phenyl)acrylate *(**15**)*

Imidazole (5.28 g, 77.51 mmol) and *tert*-butyldimethylchlorosilane (5.0 g, 33.22 mmol) were added to the crude compound **14** (2.15 g, 11.07 mmol) dissolved in dry DMF (60 mL) at 0 °C. The reaction mixture was allowed to warm up to room temperature and stirred overnight. The reaction was quenched with brine, extracted with ethyl acetate (100 mL × 3) and the combined organic layers were dried over anhydrous sodium sulfate, filtered and concentrated in vacuo. The resulting residue was purified by silica gel chromatography to afford compound **15** (4.74 g, 98%) as a white solid. ^1^H-NMR (CDCl_3_): δ 7.56 (d, *J* = 16.0 Hz, 1H), 7.01 (s, 1H), 6.99 (d, *J* = 5.6 Hz, 1H), 6.81 (d, *J* = 8.4 Hz, 1H), 6.23 (d, *J* = 16.0 Hz, 1H), 3.77 (s, 3H), 0.98 (s, 9H), 0.97 (s, 9H), 0.20 (s, 6H), 0.20 (s, 6H).; ^13^C-NMR (CDCl_3_): δ 167.7, 149.4, 147.2, 144.8, 128.0, 122.2, 121.1, 120.4, 115.4, 51.5, 25.9, 25.9, 18.5, 18.4, −4.1, −4.1; HRMS (TOF-MS): *m*/*z* calcd for C_22_H_39_O_4_Si_2_ [M + H]^+^ 423.2381. Found 423.2376.

### 3.12. Methyl 3-(3,4-bis((tert-butyldimethylsilyl)oxy)phenyl)-2,3-dihydroxypropanoate *(**16**)*

Methyl caffeate (**15**, 2.30 g, 5.43 mmol) dissolved in *tert*-butyl alcohol (20 mL) was added to a suspension of K_2_OsO_4_ (2.80 g, 7.60 mmol), K_3_[Fe(CN)_6_] (2.50 g, 7.60 mmol), K_2_CO_3_ (1.05 g, 7.60 mmol) and methanesulfonamide (10 mmol) in *tert*-butyl alcohol (40 mL) under a nitrogen atmosphere at 0 °C. The mixture was stirred for 28 h until **15** was completely consumed. The reaction was quenched with a saturated solution of sodium sulfite. The mixture was allowed to warm up to room temperature and stirred for 1 h. The reaction mixture was extracted with CH_2_Cl_2_ (100 mL × 3) and the combined organic layers washed with brine, dried over anhydrous sodium sulfate, filtered and concentrated in vacuo. The resulting residue was purified by silica gel chromatography to give dihydroxyl ester **16** (2.10 g, 85%) as a white solid. ^1^H-NMR (CDCl_3_): δ 6.91 (s, 1H), 6.81 (s, 2H), 4.85 (s, 1H), 4.30 (s, 1H), 3.77 (s, 3H), 3.12 (s, 1H), 2.70 (s, 1H), 0.99 (s, 9H), 0.98 (s, 9H), 0.19 (s, 6H), 0.19 (s, 6H); ^13^C-NMR (CDCl_3_): δ 173.2, 146.8, 146.8, 133.0, 120.8, 119.5, 119.4, 74.9, 74.3, 52.6, 25.9, 18.4, 18.4, −4.1, −4.1.

### 3.13. Methyl 3-(3,4-bis((tert-butyldimethylsilyl)oxy)phenyl)-2-hydroxypropanoate *(**17**)*

Et_3_SiH (7.78 mL, 48.83 mmol) and trifluoroacetic acid (7.26 mL, 97.7 mmol) were added to a solution of dihydroxyl ester **16** (4.46 g, 9.77 mmol) dissolved in dry CH_2_Cl_2_ (40 mL) at 0 °C. The reaction mixture was allowed to warm up to room temperature and stirred for 10 h. The reaction was quenched with a saturated aqueous solution of sodium bicarbonate and extracted with CH_2_Cl_2_ (80 mL × 3). The combined organic layers were washed with brine, dried over anhydrous sodium sulfate, filtered and concentrated in vacuo. The resulting residue was purified by silica gel chromatography to give the monohydroxy product **17** (3.70 g, 86%) as a white semi-solid. ^1^H-NMR (CDCl_3_): δ 6.76 (d, *J* = 8.0 Hz, 1H), 6.75 (s, 1H), 6.66 (d, *J* = 8.0 Hz, 1H), 4.41 (br s, 1H), 3.74 (s, 3H), 3.00 (dd, *J* = 4.4 and 14.0 Hz, 1H), 2.89 (br s, 1H), 2.88 (dd, *J* = 6.4 and 14.0 Hz, 1H), 1.01 (s, 18H), 0.21 (s, 12H).; ^13^C-NMR (CDCl_3_): δ 174.5, 146.6, 145.8, 129.3, 122.5, 122.4, 120.9, 71.4, 52.2, 39.9, 26.0, 18.4, −4.1.

### 3.14. Methyl 3-(3,4-bis((tert-butyldimethylsilyl)oxy)phenyl)-2-(2-(dimethoxyphosphoryl)acetoxy)-propanoate *(**3**)*

Dicyclohexylcarbodiimide (DCC, 2.58 g, 12.49 mmol) and a catalytic amount of 4-dimethylaminopyridine (0.19 g, 1.56 mmol) were added to a solution of the monohydroxy product **17** (3.44 g, 7.81 mmol) and 2-(dimethoxyphosphoryl)acetic acid **18** (2.10 g, 12.49 mmol) in dry CH_2_Cl_2_ (40 mL) at 0 °C under a nitrogen atmosphere, and the resulting mixture was then stirred for 1 h. The reaction was quenched with brine, extracted with CH_2_Cl_2_ (50 mL × 3) and the combined organic layers dried over anhydrous sodium sulfate, filtered and concentrated in vacuo. The resulting residue was purified by silica gel chromatography to give compound **3** (4.15 g, 90%) as a white semi-solid. ^1^H-NMR (CDCl_3_): δ 6.69 (d, *J* = 8.0 Hz, 1H), 6.64 (s, 1H), 6.60 (d, *J* = 8.4 Hz, 1H), 5.15 (dd, *J* = 4.8 and 7.2 Hz, 1H), 3.72 (d, *J* = 11.2 Hz, 3H), 3.70 (d, *J* = 11.2 Hz, 3H), 3.64 (s, 3H), 2.97 (d, *J* = 21.6 Hz, 2H), 2.94–3.00 (m, 2H), 0.93 (s, 9H), 0.92 (s, 9H), 0.14 (s, 6H), 0.13 (s, 6H).; ^13^C-NMR (CDCl_3_) : δ 169.4, 165.1 (d, *J* = 4.7 Hz), 146.7, 146.0, 128.4, 122.2, 122.2, 120.9, 74.1, 53.2, 52.2, 36.6, 32.9 (d, *J* = 135.0 Hz), 25.9, 18.4, 18.4, −4.1; HRMS (TOF-MS): *m*/*z* calcd for C_26_H_47_O_9_PSi_2_Na [M + Na]^+^ 613.2388. Found 613.2386.

### 3.15. 2-(3,4-Bis(tert-butyldimethylsilyloxy)phenyl-1-methoxycarbonyl)ethyl(E)-3-((E)-2,3,3′,4′-tetrakis(tert -butyldimethylsilyloxy)stylben-6-yl)acrylate *(**19**)*

NaH (30.9 mg, 0.77 mmol, 60% in mineral oil) was added to a solution of **3** (0.453 g, 0.77 mmol) in dry THF (4 mL) at 0 °C under a nitrogen atmosphere and the reaction mixture stirred for 30 min. Aldehyde **2** (0.427 g, 0.59 mmol) dissolved in dry THF (1.0 mL) was added slowly to the mixture and stirred for another 3 h at 0 °C. The reaction was quenched with a saturated aqueous solution of ammonium chloride, extracted with ethyl acetate (10 mL × 3) and the combined organic layers dried over anhydrous sodium sulfate, filtered and concentrated in vacuo. The resulting residue purified by silica gel chromatography to give compound **19** (0.60 g, 65%, *E*:*Z* = 77:23) as a yellow oil. ^1^H- NMR (CDCl_3_): δ 8.04 (d, *J* = 15.6 Hz, 1H), 7.18 (d, *J* = 8.0 Hz, 1H), 7.01 (d, *J* = 16.0 Hz, 1H), 6.99 (s, 1H), 6.96 (d, *J* = 8.8 Hz, 1H), 6.81 (d, *J* = 8.4 Hz, 1H), 6.73 (s, 1H), 6.72 (d, *J* = 8.8 Hz, 1H), 6.63 (d, *J* = 7.2 Hz, 1H), 6.45 (d, *J* = 16.8 Hz, 1H), 6.32 (d, *J* = 15.6 Hz, 1H), 5.25 (br s, 1H), 3.69 (s, 3H), 2.97–3.08 (m, 2H), 1.06 (s, 18H), 0.98 (s, 18H), 0.96 (s, 18H), 0.28 (s, 6H), 0.23 (s, 12H), 0.19 (s, 12H), 0.11 (s, 6H).; ^13^C-NMR (CDCl_3_): δ 169.9, 165.9, 148.3, 146.6, 146.4, 146.1, 145.5, 145.3, 144.2, 136.7, 133.0, 130.7, 128.6, 126.5, 121.9, 121.7, 121.1, 120.6, 120.4, 119.9, 119.1, 118.7, 115.0, 72.6, 51.6, 36.4, 25.7, 25.5, 25.5, 18.3, 18.0, 17.9, −3.8, −4.0, −4.0, −4.5; HRMS (TOF-MS): *m*/*z* calcd for C_63_H_108_O_10_Si_6_Na [M + Na]^+^ 1215.6450. Found 1215.6457.

### 3.16. 3-(3,4-Dihydroxyphenyl)-1-methoxy-1-oxopropan-2-y(E)-3-(2-((E)-3,4-dihydroxystyryl)-3,4-dihydroxyphenyl)acrylate *(**1**)*

Et_3_N•3HF (0.81 g, 5.02 mmol) was added to a solution of 19 (0.50 g, 0.42 mmol) in dry pyridine (4 mL) at 0 °C under a nitrogen atmosphere. The reaction mixture was stirred for 1 h and then diluted with methanol. The mixture was extracted with CH_2_Cl_2_ (10 mL × 3) and the combined organic layers washed with brine, dried over anhydrous sodium sulfate, filtered and concentrated in vacuo. The resulting residue was purified by silica gel chromatography to give (±)-methyl salvianolate A (**1**, 0.196 g, 92%) as a yellow solid. ^1^H-NMR (CD_3_OD): δ 8.00 (d, *J* = 16.0 Hz, 1H), 7.07 (d, *J* = 16.4 Hz, 1H), 7.05 (d, *J* = 8.0 Hz, 1H), 6.98( s, 1H), 6.81 (d, *J* = 7.2 Hz, 1H), 6.69 (d, *J* = 8.0 Hz, 1H), 6.68 (d, *J* = 8.4 Hz, 1H), 6.58 (s, 1H), 6.57 (d, *J* = 7.6 Hz, 1H), 6.56 (d, *J* = 16.0 Hz, 1H), 6.44 (d, *J* = 8.0 Hz, 1H), 6.21 (d, *J* = 16.0 Hz, 1H), 5.11 (dd, *J* = 5.2 and 7.2 Hz, 1H), 3.61 (s, 3H), 2.89-2.94 (m, 1H).; ^13^C-NMR (CD_3_OD): δ 170.8, 167.1, 146.9, 146.2, 145.3, 145.1, 144.7, 143.9, 143.0, 136.5, 130.0, 127.4, 127.1, 124.7, 120.6, 119.3, 119.0, 118.7, 116.0, 115.1, 115.0, 113.9, 113.5, 112.7, 73.3, 51.3, 36.5; HRMS (TOF-MS): *m*/*z* calcd for C_27_H_24_O_10_Na [M + Na]^+^ 531.1262. Found 531.1262.

## 4. Conclusions

We have accomplished the first total synthesis of the polyphenol natural product, (±)-methyl salvianolate A, using a convergent approach. The key features of the approach are the Horner–Wadsworth–Emmons reaction and the protection of multiple hydroxyl groups using silyl protecting groups, which avoid the side reactions often observed during the deprotection step. Employment of the readily removable silyl protecting group in the present approach is a practicable route to methyl salvianolate A and its derivatives. Further studies on the application of this approach to natural products containing polyphenol units are in progress and will be disclosed in due course.

## Figures and Tables

**Table 1:**
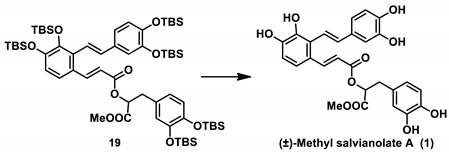
Completion of the total synthesis of (±)-methyl salvianolate A.

Entry	Reagent	Solvent	Yield (%) ^a^
1	TBAF	THF	– ^b^
2	CSA	MeOH	No reaction
3	TsOH	MeOH	No reaction
4	0.1M HCl	H_2_O	No reaction
5	2.0 M HCl	H_2_O	– ^b^
6	Cs_2_CO_3_	DMF-H_2_O (1:1)	No reaction
7	LiOH	DMF	– ^b^
8	NaOH	DMF	– ^b^
9	Et_3_N·3HF	pyridine	92%

^a^ Isolated yield after silica gel column chromatography. ^b^ Starting material was completely consumed, but only a trace amount of the target product was isolated using silica gel column chromatography with a large amount of 1 not separable from the crude mixture containing silicide.

## Supplementary Materials

NMR spectra for all synthetic compounds are available online.
