# Supplementary material for: The First Total Synthesis of (±)-Methyl Salvianolate A Using a Convergent Strategy
Source: Molecules. 2019 Mar 12;24(5):999. doi: 10.3390/molecules24050999 (PMC6429350; doi:10.3390/molecules24050999)
Supplement: Supplementary file 1 [file molecules-24-00999-s001.pdf]

Supplementary Material

The First Total Synthesis of (±)-Methyl Salvianolate A Using a Convergent Strategy

Bo Wang [1,†], Liping Wang [2,†], Ying Peng [2], Yiying Pang [2], Hesheng Xiao [2], Xiaoji Wang [2,*] and Shuangping Huang [3,4,*]

[1] School of Chemistry and Chemical Engineering, Jiangxi Science and Technology Normal University, Nanchang 330013, China; wbo0728@163.com
[2] School of Life Science, Jiangxi Science and Technology Normal University, Nanchang 330013, China; wlping92@163.com (L.W.); dapengpeng@163.com (Y.P.); 13667080401@163.com (Y.P.); xiaohes93@163.com (H.X.);
[3] School of Pharmacy, Jiangxi Science and Technology Normal University, Nanchang 330013, China
[4] College of Biomedical Engineering, Taiyuan University of Technology, Taiyuan 030024, China
* Correspondence: 2012207455@tju.edu.cn (X.W.); hsping02@gmail.com (S.H.); Tel.: +86-791-8380-5358 (S.H.)
† These two authors contributed equally to this work.

^{1}H-NMR spectrum of **8** in $CDCl_3$

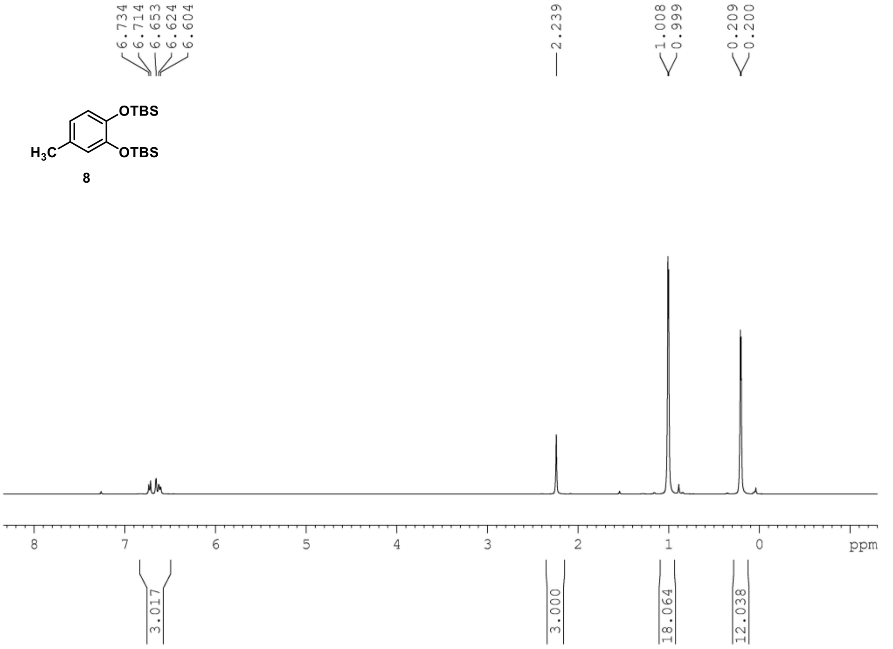

^{1}H-NMR spectrum of **4** in $CDCl_3$

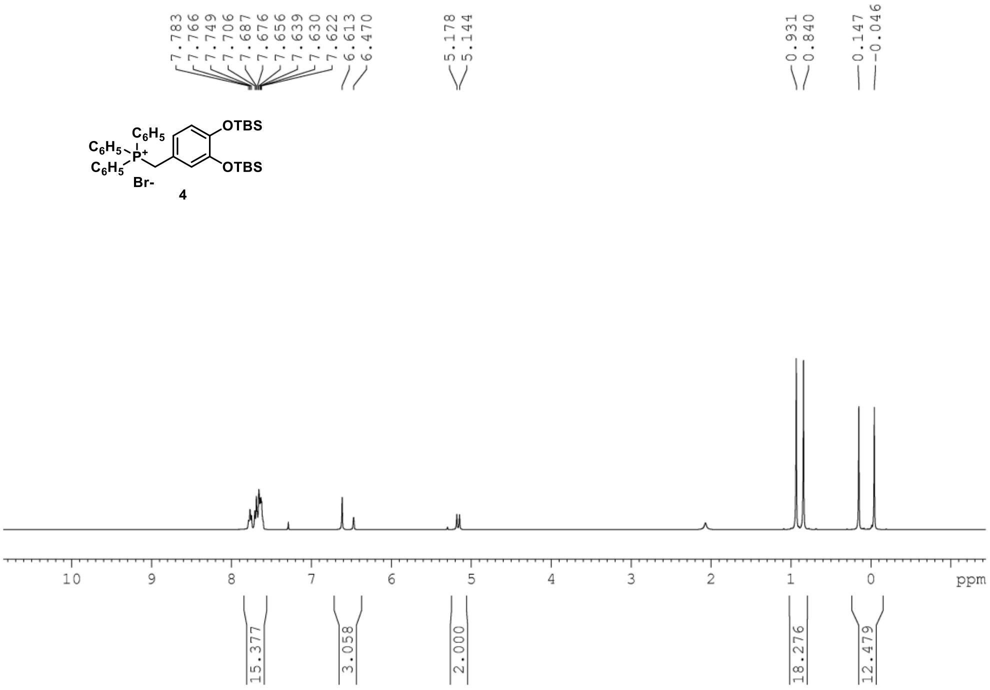

^{1}H- and ^{13}C-NMR spectra of **10** in $CDCl_3$

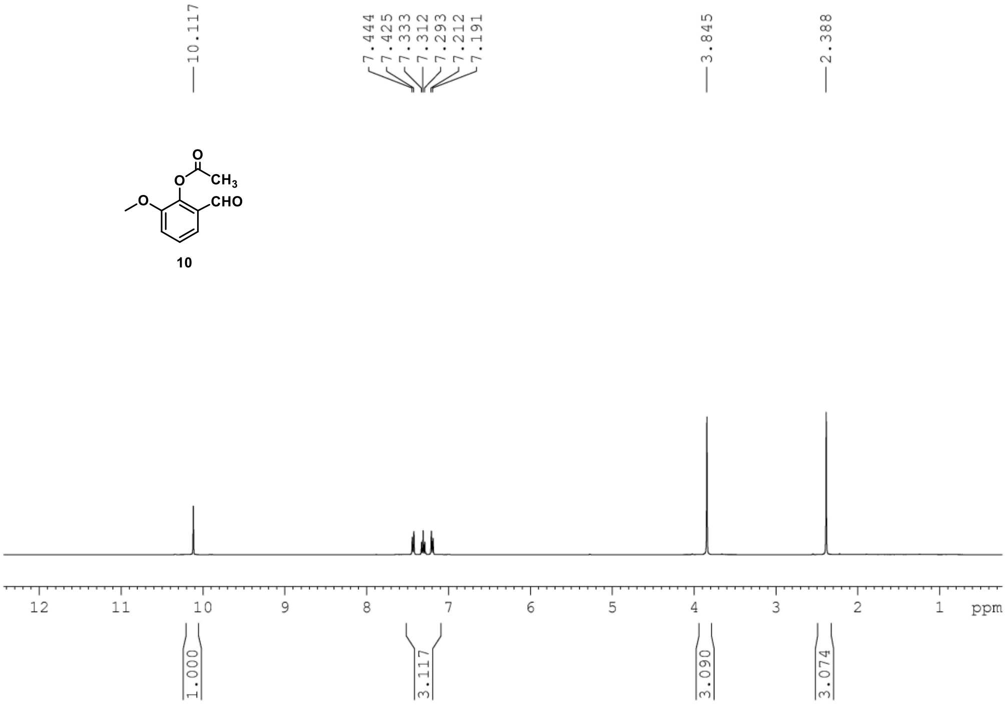

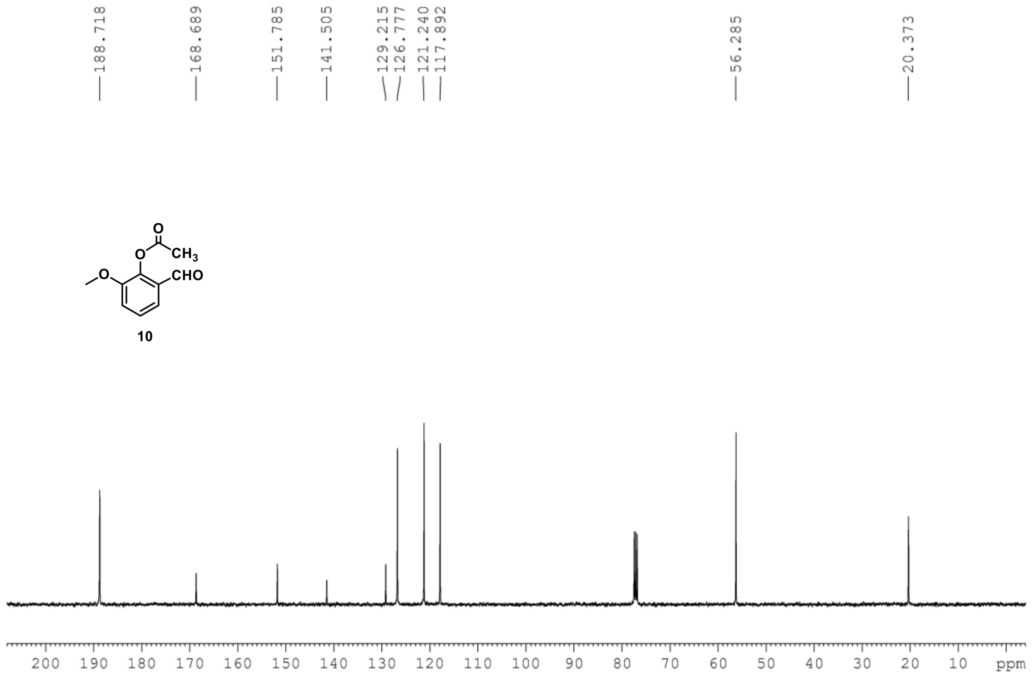

^{1}H- and ^{13}C-NMR spectra of **11** in $CDCl_3$

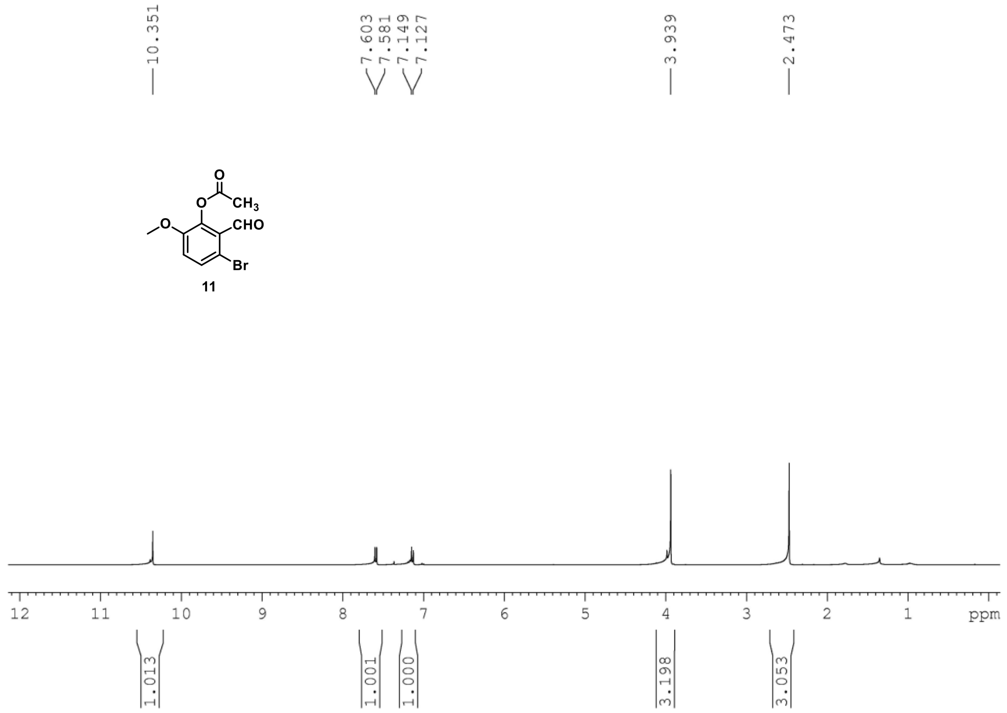

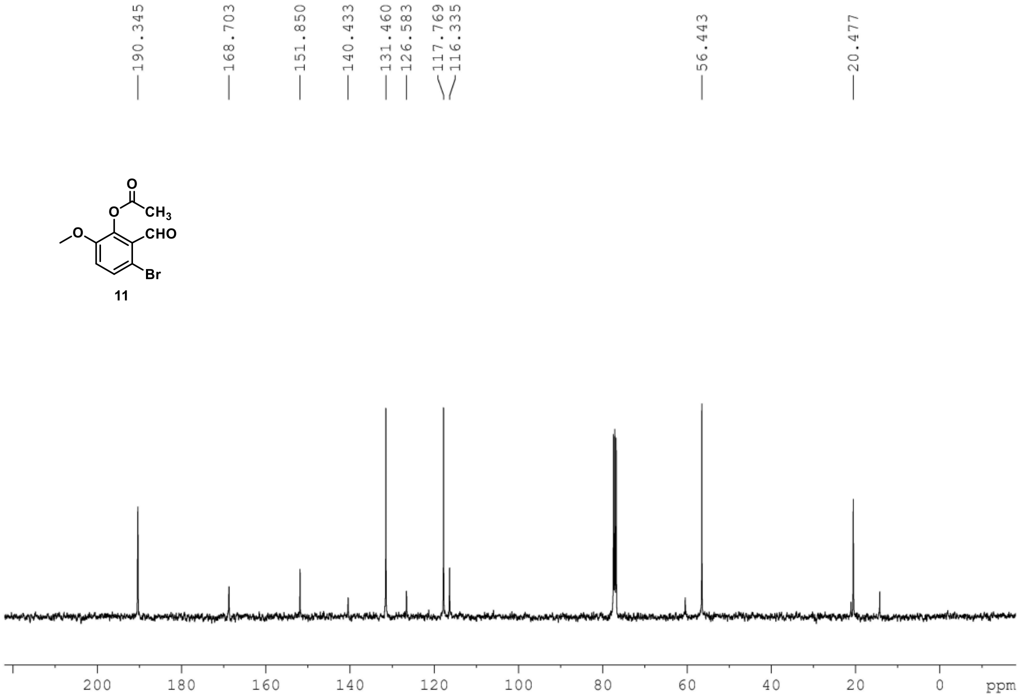

^{1}H- and ^{13}C-NMR spectra of **12** in $CDCl_3$

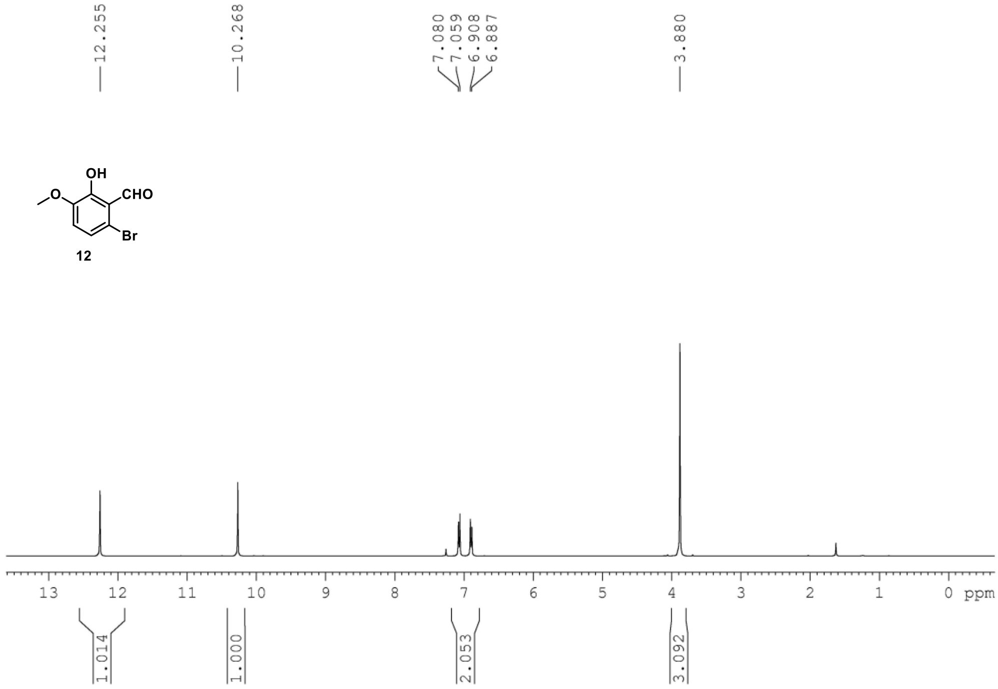

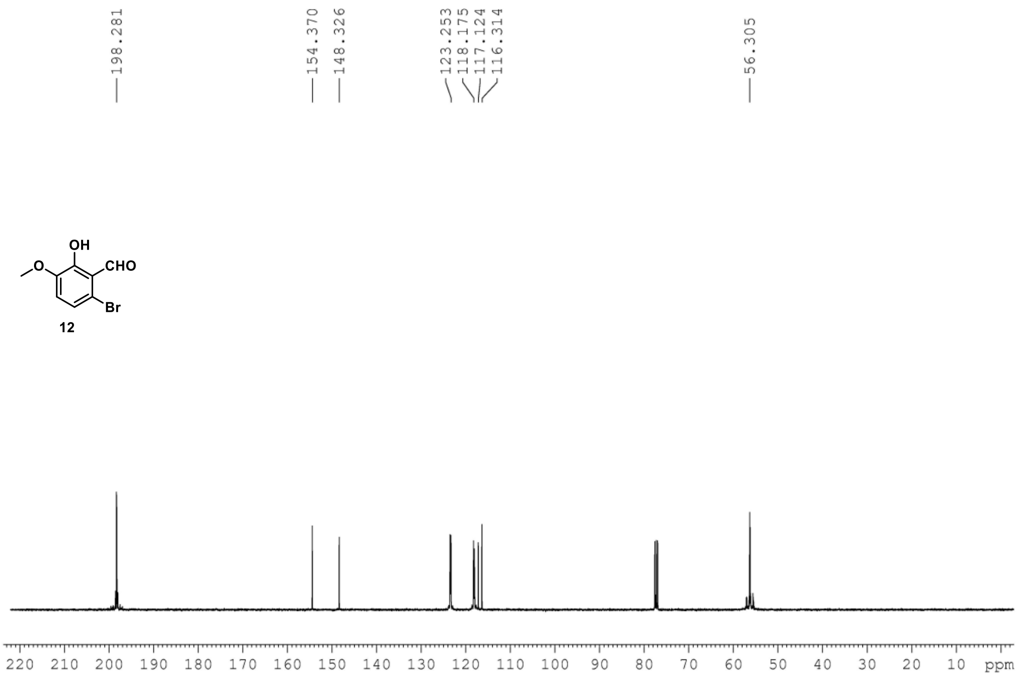

^{1}H- and ^{13}C-NMR spectra of **5** in $CDCl_3$

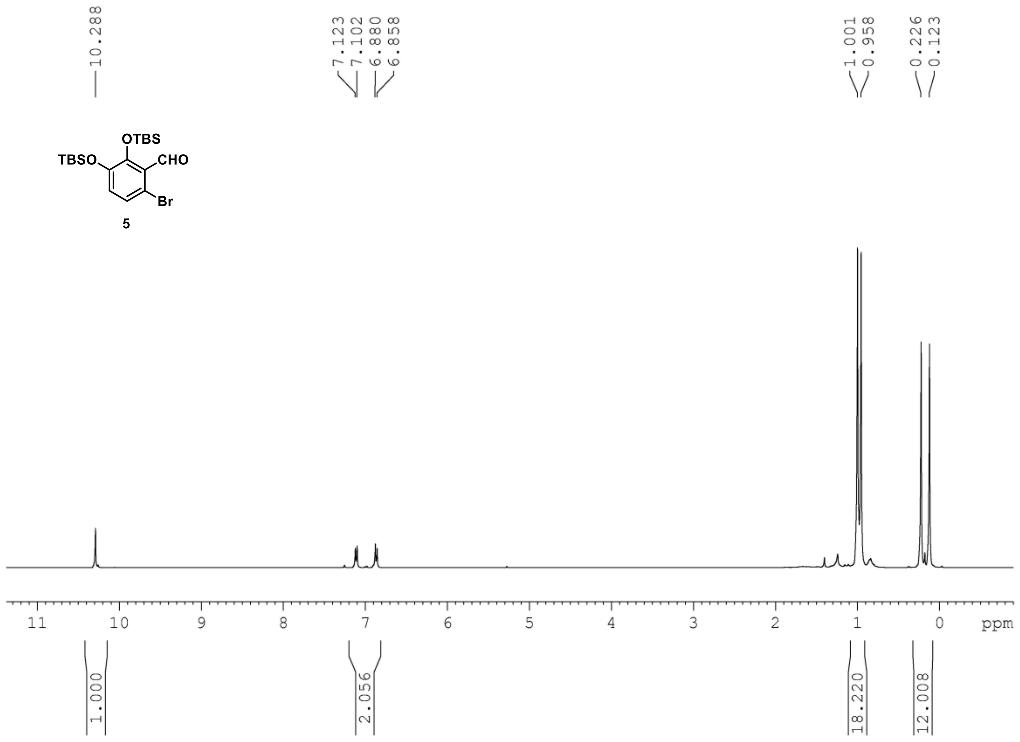

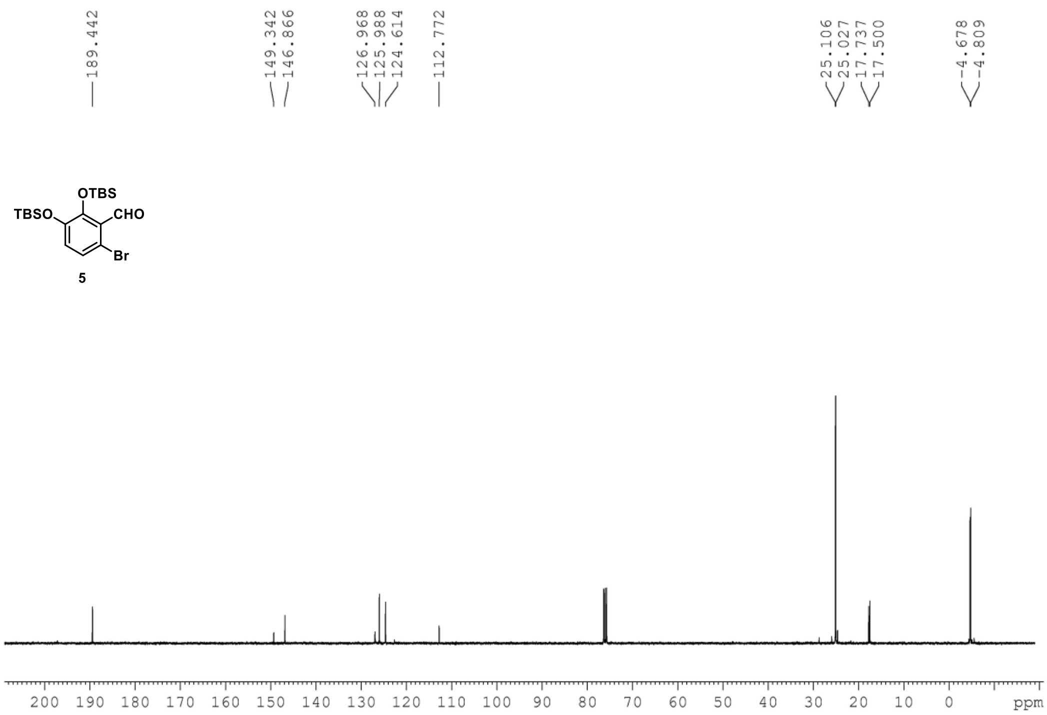

^{1}H- and ^{13}C-NMR spectra of **13** in $CDCl_3$

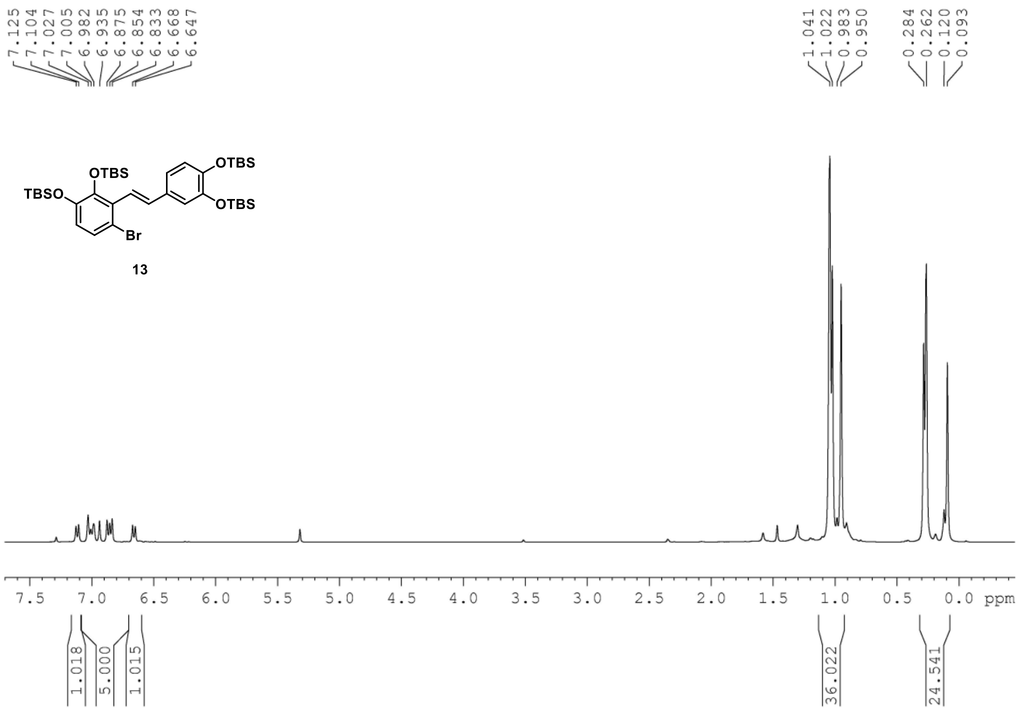

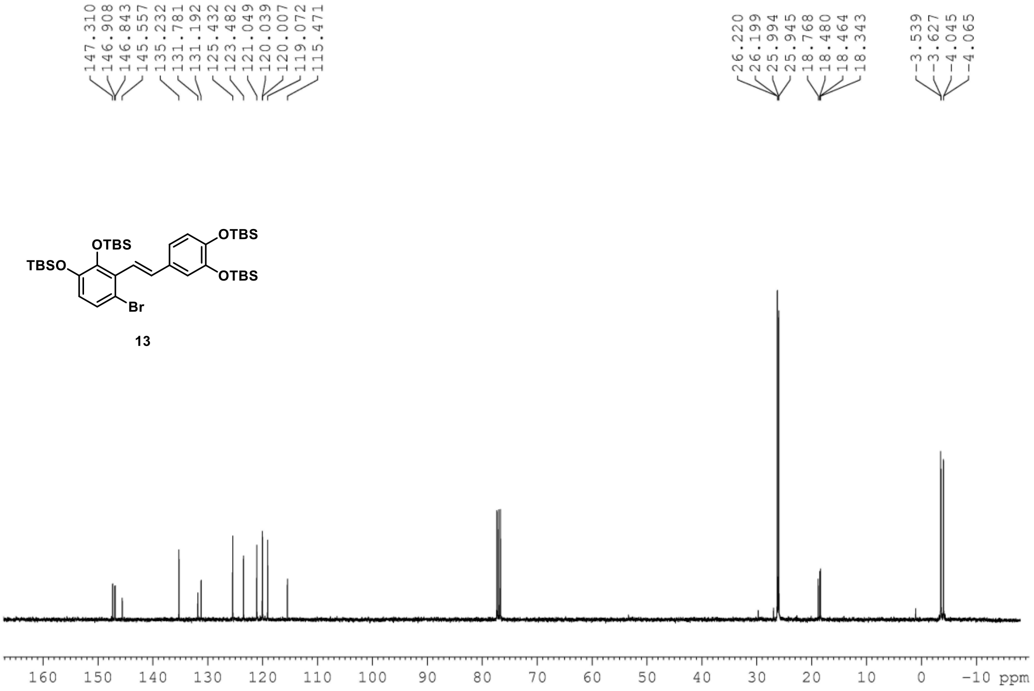

^{1}H- and ^{13}C-NMR spectra of **2** in $CDCl_3$

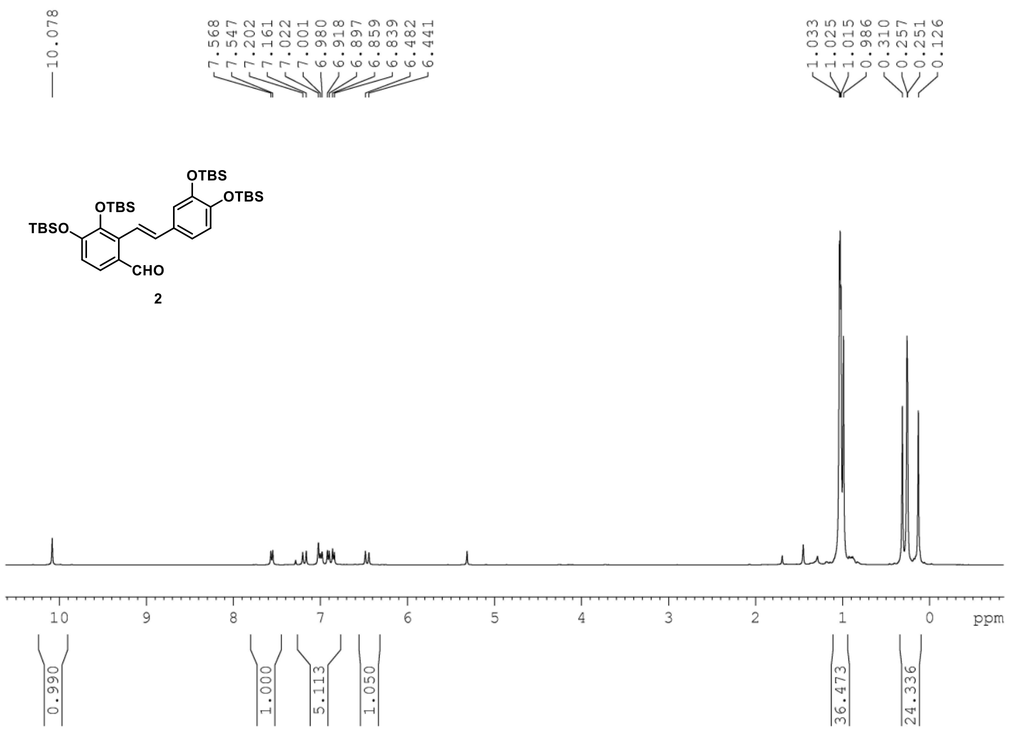

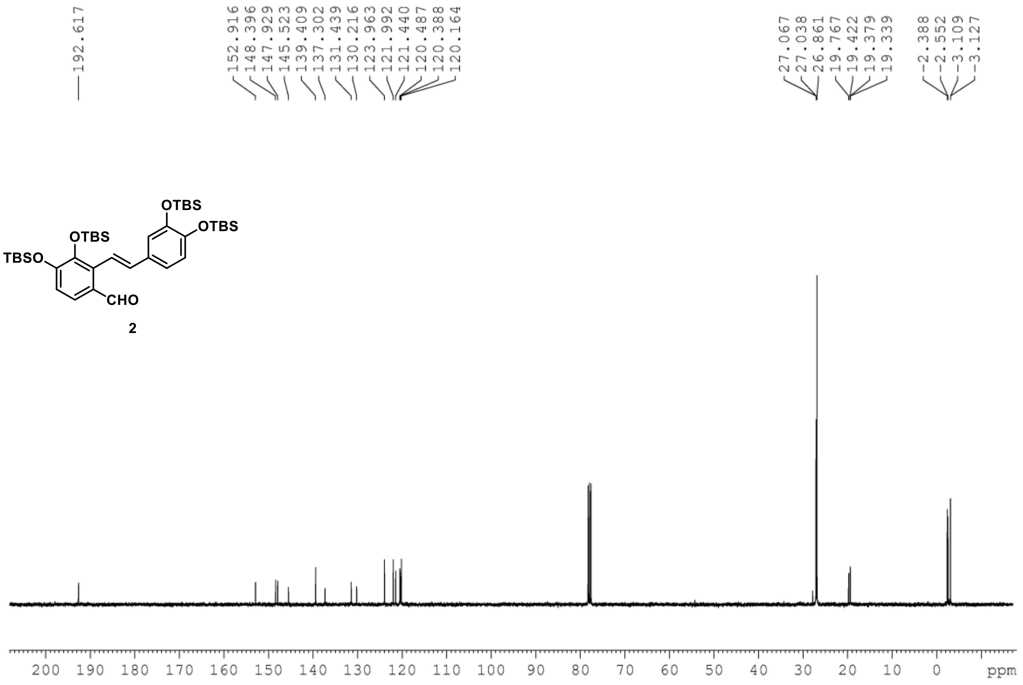

^{1}H- and ^{13}C-NMR spectra of **14** in CD_3OD

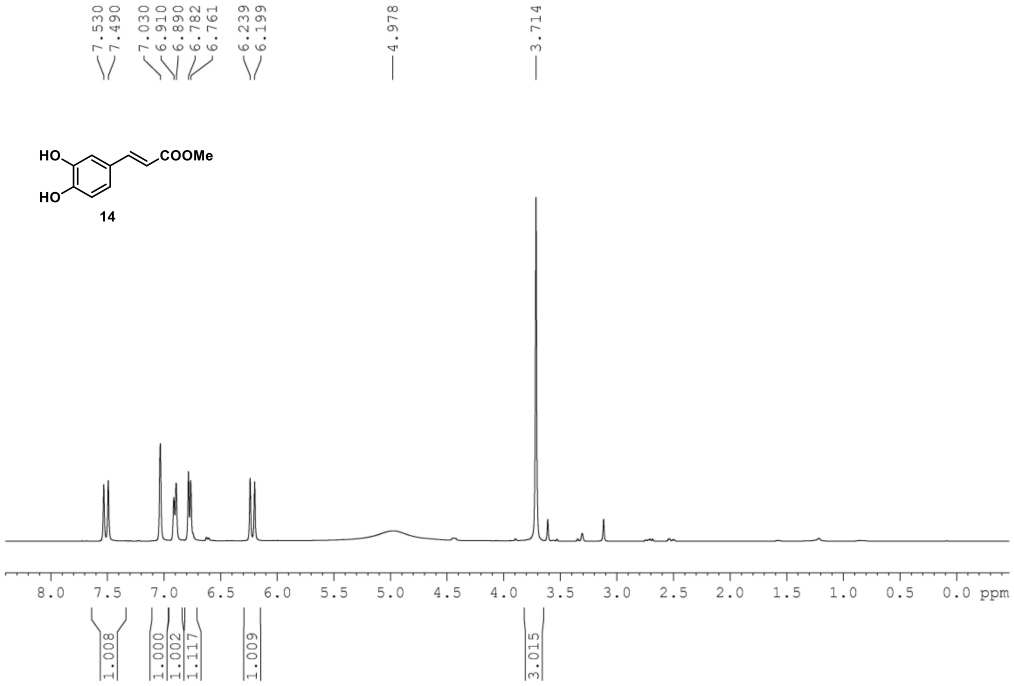

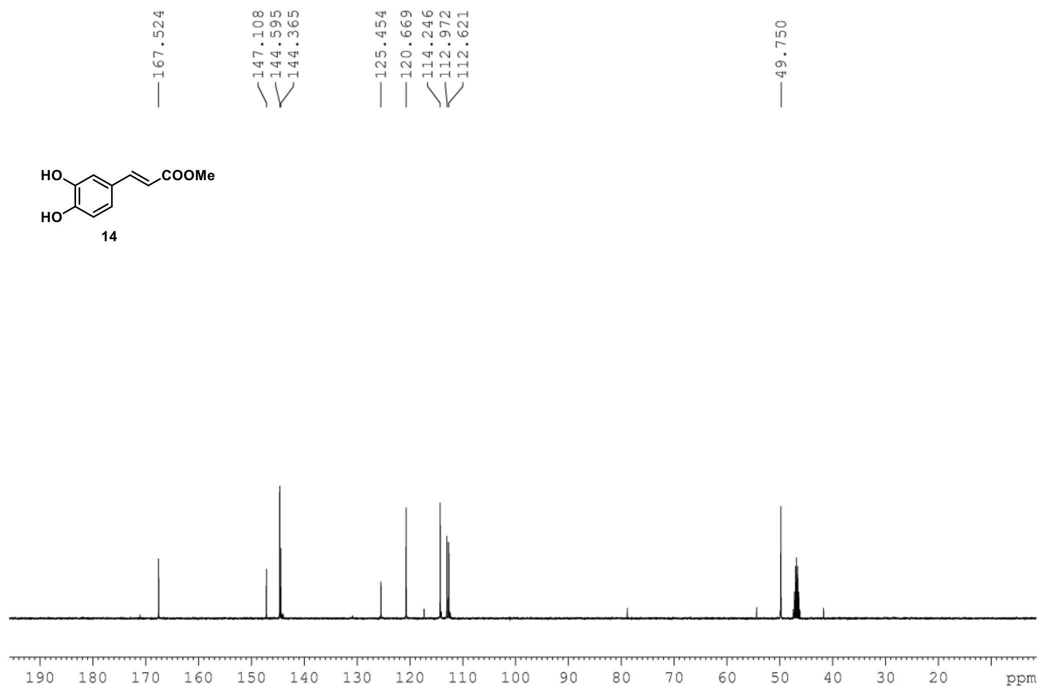

^{1}H- and ^{13}C-NMR spectra of **15** in $CDCl_3$

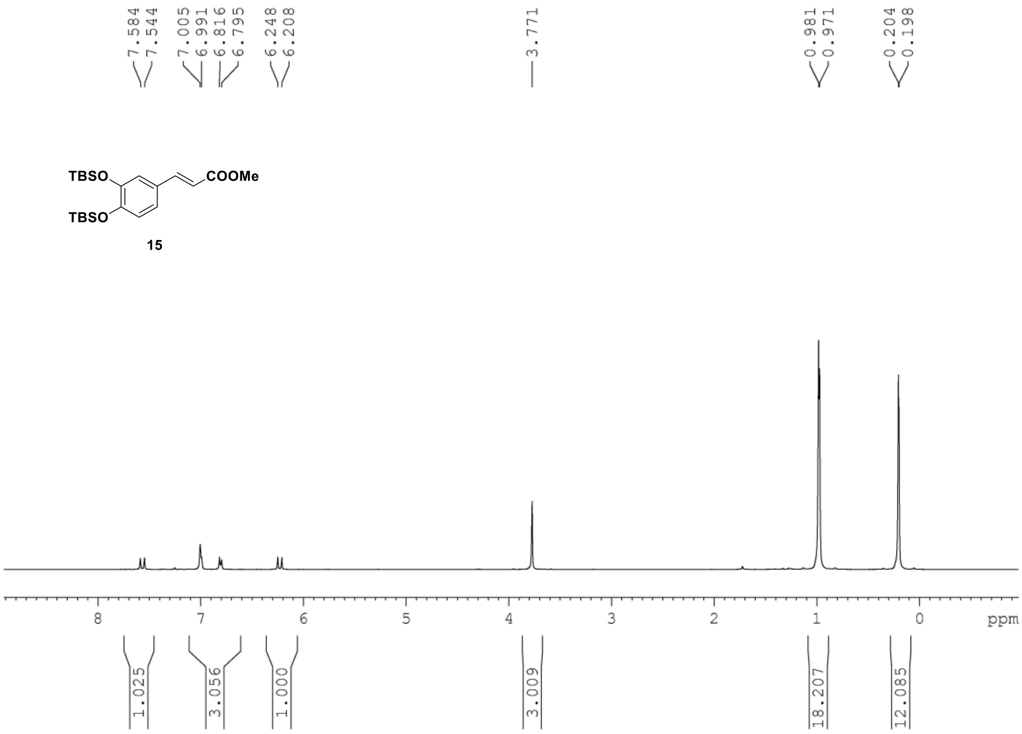

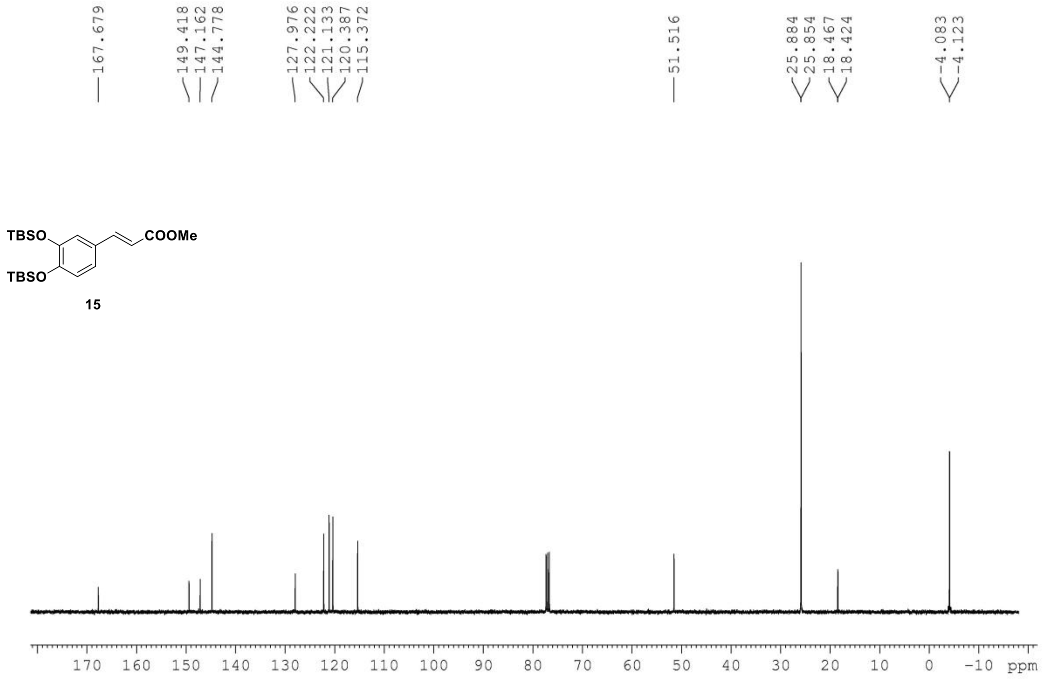

^{1}H- and ^{13}C-NMR spectra of **16** in $CDCl_3$

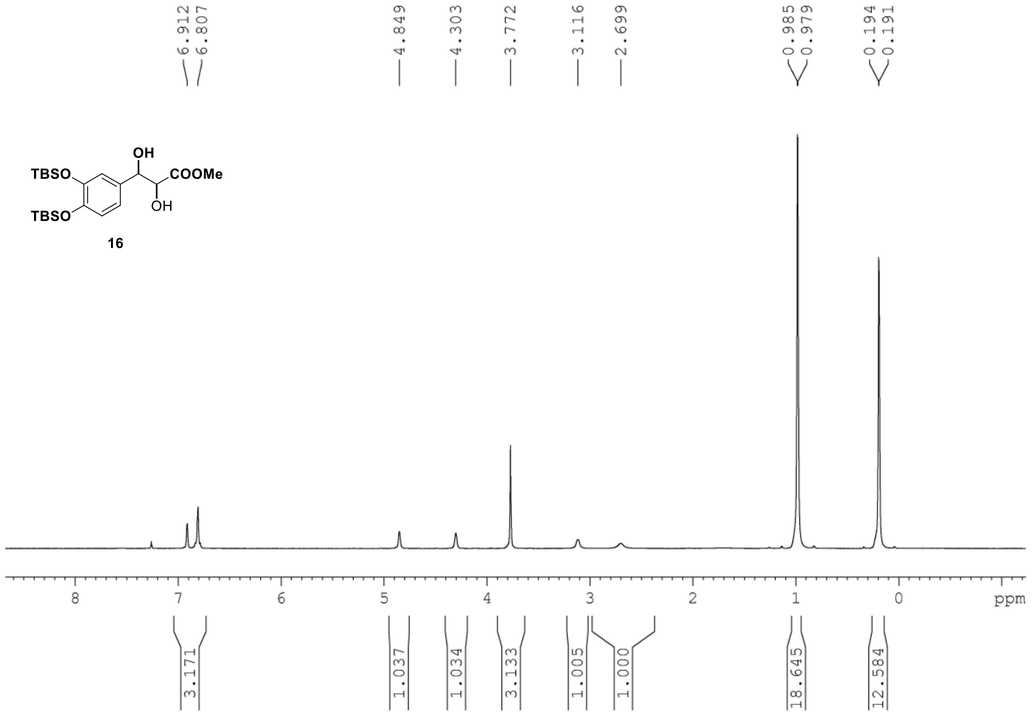

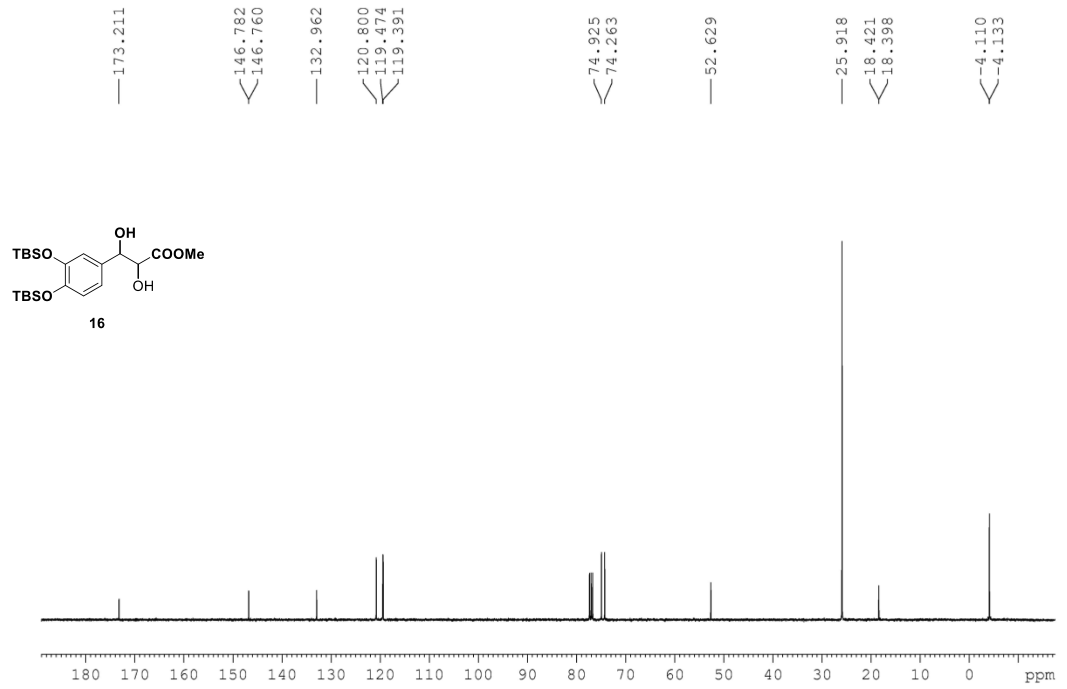

^{1}H- and ^{13}C-NMR spectra of **17** in $CDCl_3$

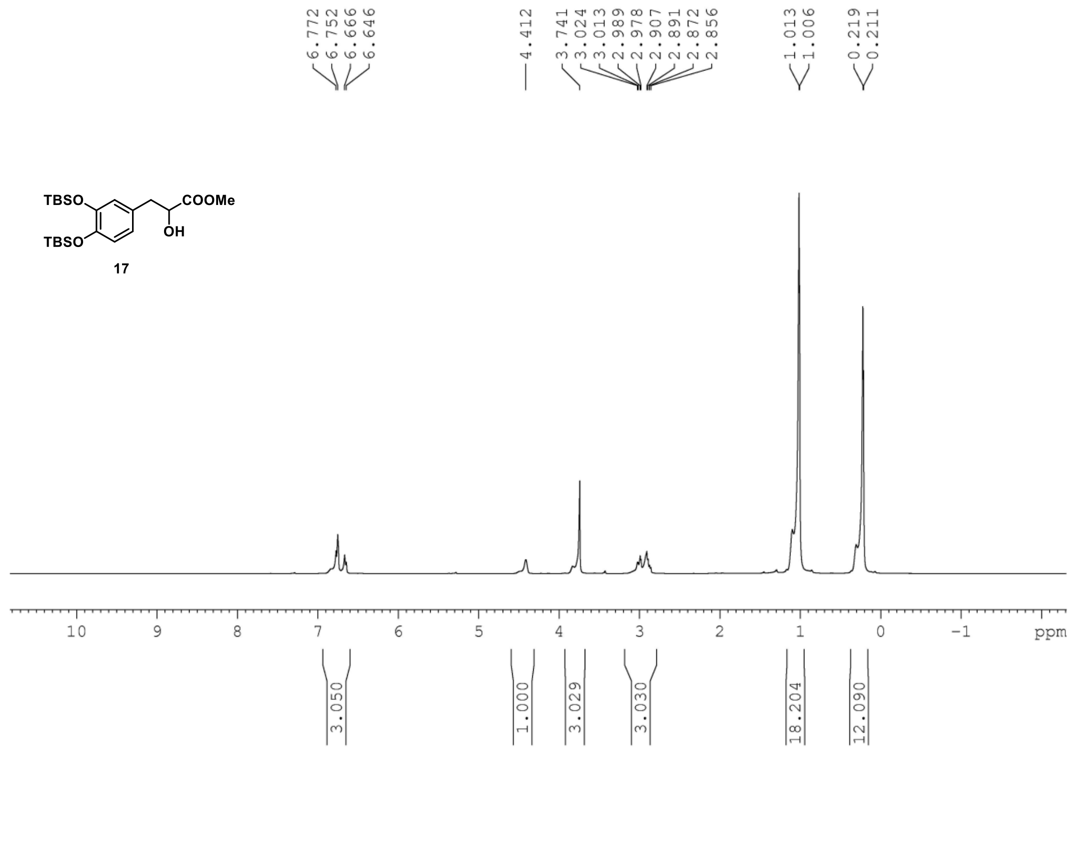

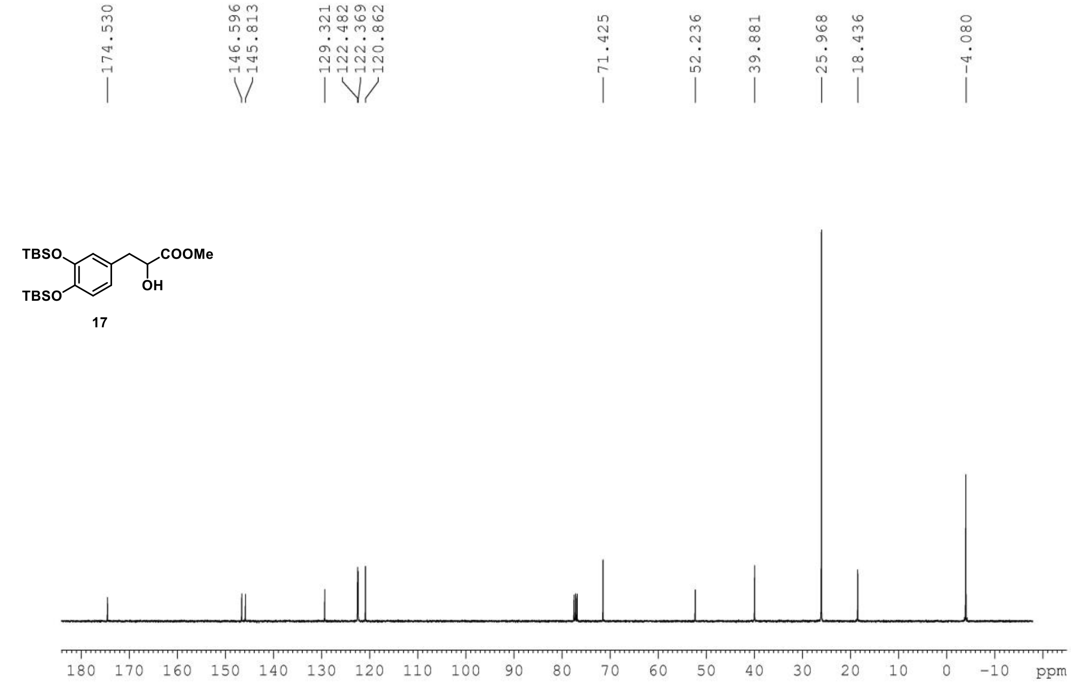

^{1}H- and ^{13}C-NMR spectra of **3** in $CDCl_3$

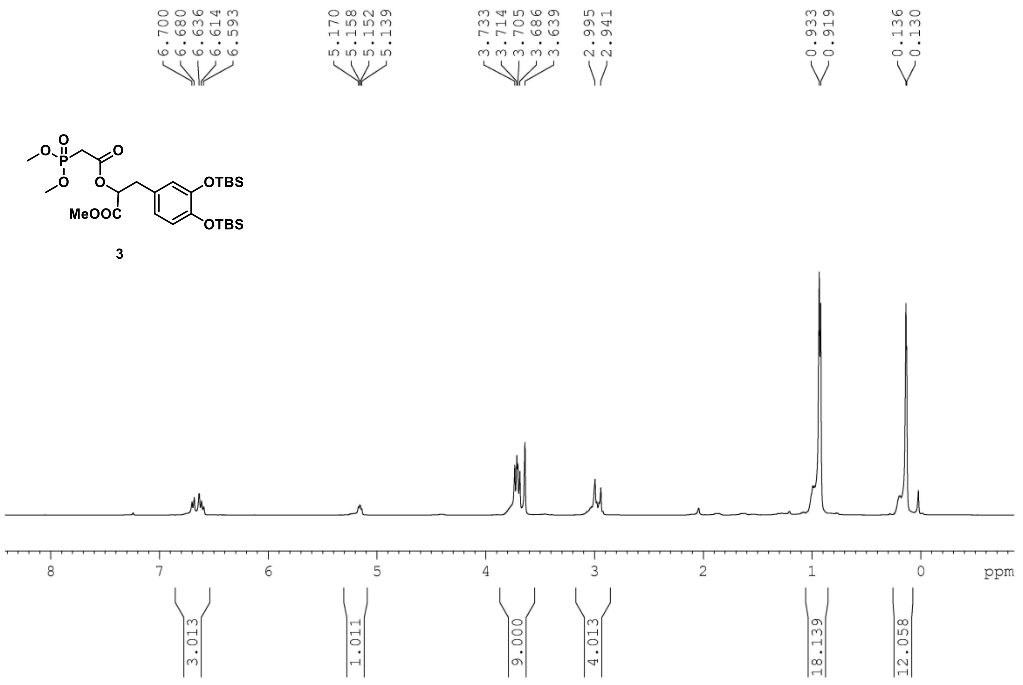

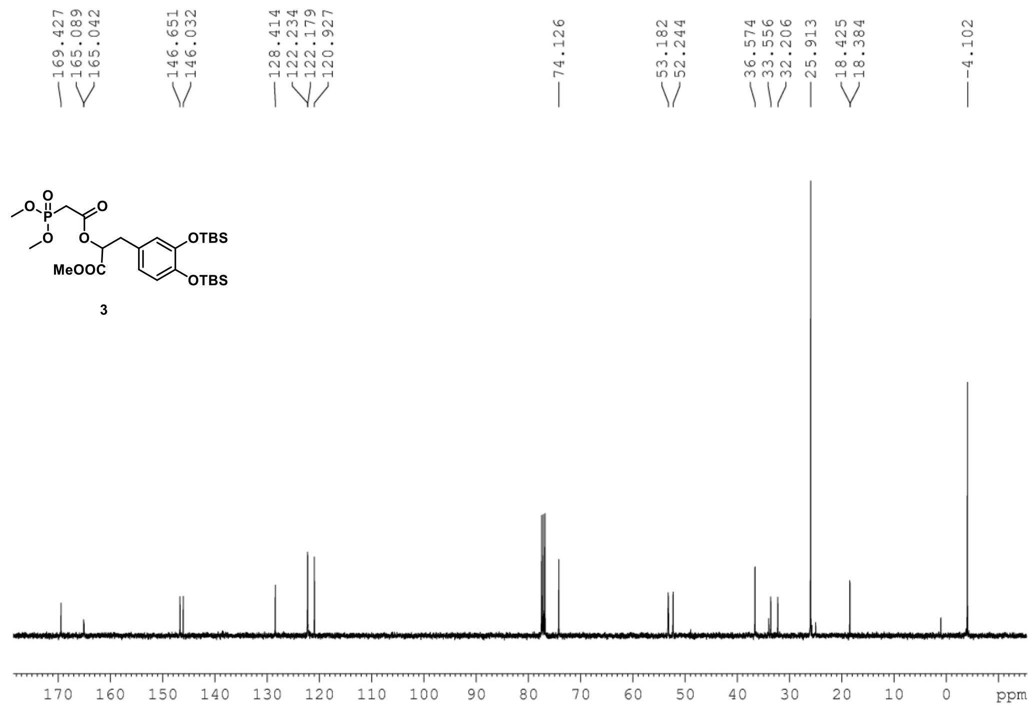

^{1}H- and ^{13}C-NMR spectra of **19** in $CDCl_3$

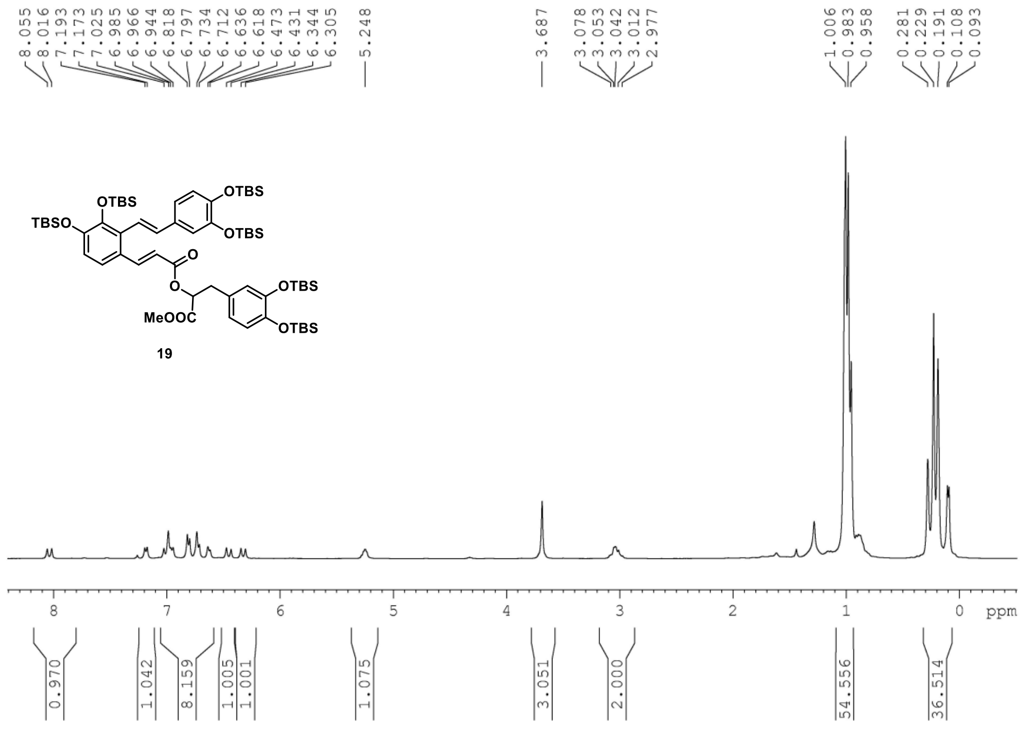

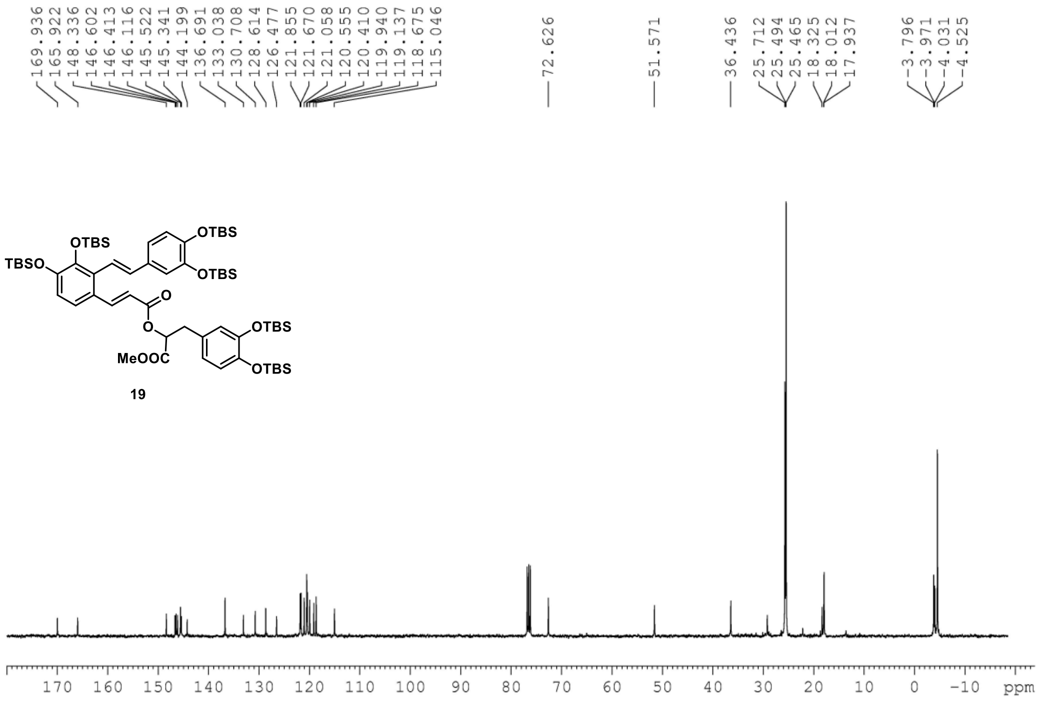

^{1}H- and ^{13}C-NMR spectra of **1** in CD_3OD

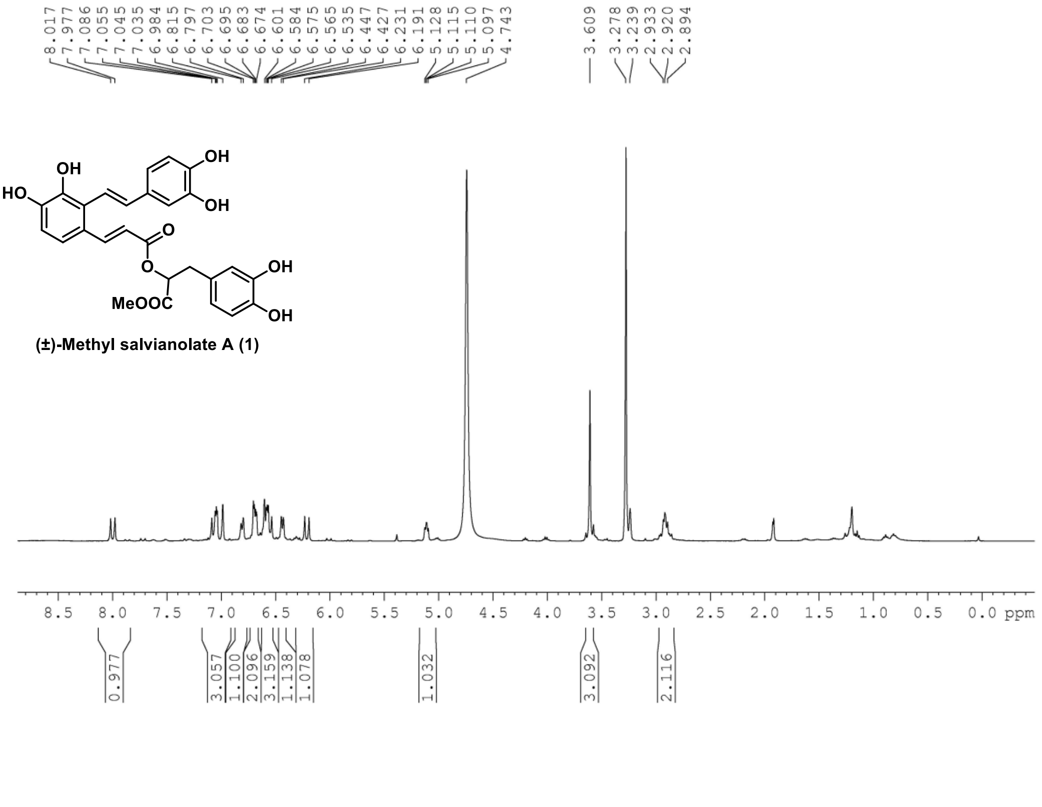

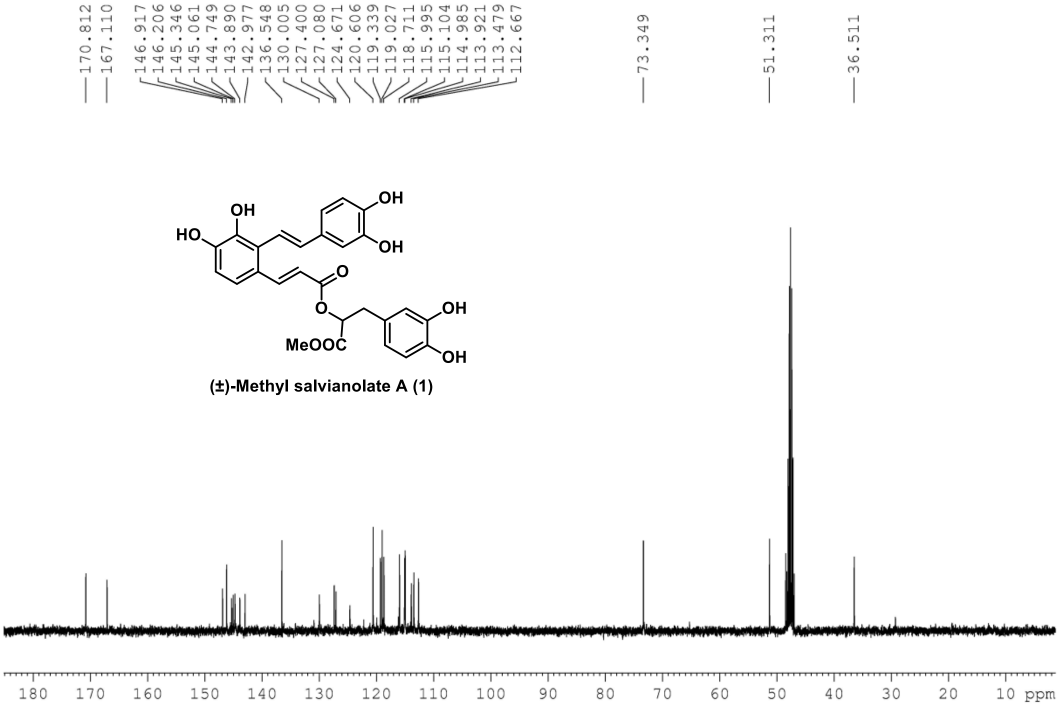

(±)-Methyl salvianolate A (1)
